# Genome-wide and expression analysis of B-box gene family in pepper

**DOI:** 10.1186/s12864-021-08186-w

**Published:** 2021-12-06

**Authors:** Jing Ma, Jia-xi Dai, Xiao-wei Liu, Duo Lin

**Affiliations:** Engineering Laboratory of Genetic Improvement of Horticultural Crops of Shandong Province, Key laboratory of horticultural plant genetic improvement and breeding of Qingdao, College of Horticulture, Qingdao Agricultural University, 700 Changcheng Road, Qingdao, 266109 China

**Keywords:** BBX, Pepper, Phylogenetic relationships, Subcellular localizations, Gene expression patterns

## Abstract

**Background:**

BBX transcription factors are a kind of zinc finger transcription factors with one or two B-box domains, which partilant in plant growth, development and response to abiotic or biotic stress. The BBX family has been identified in Arabidopsis, rice, tomato and some other model plant genomes.

**Results:**

Here, 24 *CaBBX* genes were identified in pepper (*Capsicum annuum* L.), and the phylogenic analysis, structures, chromosomal location, gene expression patterns and subcellular localizations were also carried out to understand the evolution and function of *CaBBX* genes. All these CaBBXs were divided into five classes, and 20 of them distributed in 11 of 12 pepper chromosomes unevenly. Most duplication events occurred in subgroup I. Quantitative RT-PCR indicated that several *CaBBX* genes were induced by abiotic stress and hormones, some had tissue-specific expression profiles or differentially expressed at developmental stages. Most of CaBBX members were predicated to be nucleus-localized in consistent with the transient expression assay by onion inner epidermis of the three tested CaBBX members (CaBBX5, 6 and 20).

**Conclusion:**

Several *CaBBX* genes were induced by abiotic stress and exogenous phytohormones, some expressed tissue-specific and variously at different developmental stage. The detected CaBBXs act as nucleus-localized transcription factors. Our data might be a foundation in the identification of *CaBBX* genes, and a further understanding of their biological function in future studies.

**Supplementary Information:**

The online version contains supplementary material available at 10.1186/s12864-021-08186-w.

## Introduction

In plant, transcription factors (TFs) are a kind of proteins that play an important part in physiological and biochemical processes by regulating the downstream gene transcription. There are usually four main domains for TF structure construction, all of which required for the functional process: DNA binding site, transcription activation domain, oligomerization site, and nuclear localization signal [1]. Among them, the study of B-box (BBX) zinc finger family is a growing area in recent years.

The BBX transcription factors in plants usually carry one or two B-box domains (CX_2_CX_8_CX_7_CX_2_CX_4_HX_8_H) in the N-terminal region, in which the conserved Cysteine (C) and Histidine (H) residues are predicted to be involved in protein-protein interactions [2]. The conserved B-box domains consisting of 40 amino acids was widely found in more than 1500 proteins of multicellular species and some unicellular eukaryotes [3]. Also, some plant BBX proteins are characterized containing an additional highly conserved CCT (CONSTANS, CO-like, and TIMING of CAB1) domain in the C-terminus [4, 5], which play an essential role in transcriptional regulation and nuclear transport [6, 7]. There are 32 BBXs found in *Arabidopsis*, named AtBBX1 ~ 32 [2]. These AtBBX proteins were divided into five structure groups (Groups I ~ V) depending on the number and sequence features of the B-box domain or the presence of a CCT domain [2, 8, 9]. Both Group I (AtBBX1 ~ 6) and II (AtBBX7 ~ 13) proteins possess two B-box domains and one CCT domain, with some differences at consensus sequences of second B-box domain between Group I and II [8]. AtBBX14 to AtBBX17 belonging to Group III had a single B-box domain in association with a CCT domain; the AtBBX members of structure group IV (AtBBX18 ~ 25) contain two B-box domains without CCT domain; while Group V proteins (AtBBX26 to AtBBX32) only had a single B-box domain [2]. Additionally, the BBX TFs were identified in several other model plants in recent years, such as 30 members in rice, 29 in tomato, 64 in apple, 39 in pear, and so on [10–14]. And the BBX TFs identified in all these model plants were classified into five groups in the same cases with *Arabidopsis* BBX members.

In plants, B-box (BBX) proteins are well-known to be involved in plant development, especially light, circadian signaling and flowering. *CO/AtBBX1* was the first discovered BBX protein, a core component which can promote flowering under long-day condition [15, 16]. Other two BBX proteins, *BBX2* and *BBX3* were investigated to be less influenced on flowering time, but overexpression of *BBX2* gene showed a decreasing duration of two specific circadian rhythms in Arabidopsis [17]. BBX21 (also known as SALT TOLERANCE HOMOLOG 2) were identified as a regulator of several *ABA INSENSITIVE* (*ABI*) genes and directly activates *ELONGATED HYPOCOTYL 5* (*HY5*) in ABA control of seed germination, which is targeted by COP1 for 26S proteasome-mediated degradation in *Arabidopsis* [18–20]. AtBBX28 was found that interact with both HY5 and COP1 at its C-terminal portion resulting in negatively regulates photomorphogenic development [21]. Besides, HY5 negatively regulated BBX30 and BBX31 by directly binding to the G-box cis-element present in their promoters, negatively regulate photomorphogenesis in *Arabidopsis* [22]. BBX4 is a key component involved in the phyB (Phytochrome B)-PIF3 (PHYTOCHROME INTERACTING FACTOR 3) regulatory module. phyB directly interacts with BBX4 and positively controls the level of BBX4 protein in red light. And BBX4 repressed the transcriptional activation activity of PIF3 by directly interacting with PIF3, thereby promoting photomorphogenesis [23]. And BBX11-BBX21-HY5 can positively regulate photomorphogenesis in the response to light during normal development [24]. Besides, several BBX are identified to be involved in flowering by positively and negatively regulating the *CO* (*CONSTANS*) and *FT* (*Flowering Locus T*) genes expression [25–29]. In rice, *OsCO3* possessing a single B-box and CCT domain functions as a negative FT-like genes regulator which delays flowering time under SD (short day) conditions [30]. *OsCLO4* showed a represses flowering under SD and LD (long day) conditions [31]. And *Hd1* (*OsBBX18*) containing two B-box motifs and one additional CCT domain, promote flowering under SD conditions and inhibit under LD conditions [4, 32]. Nevertheless, several BBXs in other plants, such as barely (*Hordeum vulgare*), beetroot (*Beta vulgaris*), chrysanthemum (*Chrysanthemum morifolium*), and grape (*Vitis vinifera*), also play an important role in regulation of flowering [4, 30, 33].

In addition, *BBX* family genes have shown their roles in mitigating abiotic stresses. The salt tolerance protein (STO, *AtBBX24*) was first identified to trigger the salt tolerance activities in yeast cells [34], which can enhance *Arabidopsis* root growth under salt stress treatment [35]. STO negatively regulated a wide range of stress-related genes [36], which can also interact with CLONE EIGHTY-ONE/RADICAL INDUCED CELL DEATH1 (CEO/RCD1) [37, 38]. AtBBX18 was detected to be a negative regulator both in photomorphogenesis and heat tolerance in *Arabidopsis* [39]. In rice and tomato, most promoters of the *OsBBX* and *SlBBX* genes contain at least one stress-responsive cis-element (ARE, Wbox, GC-motif, Box-W1, HSE, and MBS). Through the expression analysis under biotic or abiotic stress, the expression levels of most of BBX family members in the rice and tomato were significantly changed under most treatments, indicating that these genes were induced by biotic or abiotic stress [10, 11]. In *Chrysanthemum*, *CmBBX24* not only play a part in delaying flowering time, but also enhance cold and drought tolerance in plant [33]. Besides, overexpression of *VvBBX32* can increase cold tolerance in transgenic *Arabidopsis* plants [40]. Recently, an apple B-box protein BBX37 was identified that regulates jasmonic acid mediated cold tolerance through the JAZ-BBX37-ICE1-CBF pathway [41]. Although the studies of BBX transcription factors are increasing rapidly in previous years, there are few studies about *BBX* genes in pepper [42]. And the whole genome sequencing of *Capsicum annuum* L. makes it possible for analyzing deeply in the *BBX* gene family of pepper [43, 44].

Pepper (*Capsicum annuum* L.) is a dominant vegetable species belonging to Solanaceae family cultivated all over the world. And in recent one or two years, it has overtaken tomato, with the cultivated area in first place in the world. However, the vegetative and reproductive growth of pepper was negatively affected by biotic and abiotic stresses such as salt, cold, heat, drought, diseases and insect pests. And BBX are thought to play important roles in plant abiotic and biotic stress responses, thus the study of CaBBX TFs in these molecular mechanisms is necessary to determine the biological processes involved in the multiple regulatory of abiotic tolerance. In this study, 24 CaBBX members were identified in pepper. And we also performed the gene structure, phylogenetic relationships, chromosome localization, subcellular localizations and their expression patterns under various abiotic stresses and hormones treatments in pepper.

## Materials and methods

### Identification of BBX family genes in pepper

We firstly obtained the conserved B-box domain (PF00643) based on a Hidden Markov Model (HMM) from the Pfam database (Pfam 32.0, http://pfam.xfam.org/). Then the HMM profile of the B-box domain was utilized to do BLASTP search by using HMMER 3.2 in pepper genome databases with an expected value (*e*-value) cut-off of 0.01 [43, 44]. Afterwards, the putative CaBBX proteins obtained were confirmed for the presence of the B-box domain by the SMART (http://smart.embl-heidelberg.de/) and Pfam (http://pfam.xfam.org/) searches and InterProscan (http://www.ebi.ac.uk/interpro/search/sequence-search) programs. In addition, the isoelectric point (pI) and molecular weight (kDa) of the obtained CaBBX proteins were determined by using the ExPASy proteomics server (https://web.expasy.org/) [45].

### Phylogenetic analysis and sequence alignment

The BBX sequences of tomato were obtained from the NCBI database (http://www.ncbi.nlm.nih.gov/). Multiple sequence alignments of CaBBX proteins were carried out with the ClustalX program (Version 2.1) [46]. The p-distance-based phylogenetic tree was constructed with the neighbor-joining algorithm in MEGA (version 7.0) with a bootstrap value of 1000 [47].

### Domains, motif structure and gene structure analysis

Domains were identified with Conserved Domain Database (CDD) in NCBI (https://www.ncbi.nlm.nih.gov/cdd). MEME Suite was used to determine all motifs in the CaBBX protein sequences [48]. Analysis was performed using the following parameters: number of repetitions, if any; optimum width of the motif, 6–50; and maximum number of motifs, 8. And the intron and exon were determined by CDS and genomic information in pepper Genome Database (http://pepperhub.hzau.edu.cn/). All these structures were visualized by TBtools [49].

### Chromosomal location and duplication analysis of *CaBBX*s

The identified *CaBBX* gene annotations and their chromosomal locations were retrieved from genome annotations downloaded from the Pepper Genome Database (http://pepperhub.hzau.edu.cn/) according to the gene ID. The exact location of genes on chromosomes was drawn by using TBtools. Duplication analysis was also constructed by using TBtools [49].

### Plant materials, growth condition, hormone and stress treatments to plants

Pepper seeds (‘Qingnong dried No.2’) were obtained from the State Key Laboratory of Crop Genetics and Germplasm Enhancement in Qingdao Agricultural University. This cultivar was selected by researchers at Qingdao Agricultural University (Qingdao, China), the Qingdao Seed Station, and Dezhou Academy of Agricultural Sciences. Additionally, it was approved by the Shandong Variety Examination and Approval Committee in 2015(deposition number: 2015–057-1). At first, the seeds were germinated in light incubator at 28 °C. Three days later, the germinated seeds were transplanted into pot in a growth chamber with a photoperiod of 14 h of light and 10 h of darkness at 28 °C/21 °C. Six-leaf seedlings were used to treat with 100 μM Abscisic acid (ABA), 100 μM Methyl jasmonate (MeJA), 100 μM Salicylic acid (SA), 10% polyethylene glycol-6000 (PEG-6000), and 100 mM NaCl. High and low temperature were applied by placing seedlings in 38 and 4 °C growth chamber, respectively. The leaf tissues were harvested at 0, 3, 6 and 12 h post various treatments. And all collected samples were immediately frozen in liquid nitrogen, and then stored at − 80 °C for RNA isolation. All samples were collected in triplicate from each of the sampling points. Besides, the samples of root, stem, leaf, flower, fruit and seed were harvested to investigate the tissue-specific expression.

### Total RNA isolation and cDNA synthesis

Total RNA was isolated from plant materials using a total RNA kit (Tiangen, Beijing, China) according to the manufacturer’s instruction. A total of 1 μg of RNA of each sample was used for first-strand cDNA synthesis using M-MLV reverse transcriptase according to the manufacturer’s protocols (TaKaRa, Dalian, China). cDNA was diluted 20-fold for qRT-PCR analysis.

### Quantitative real-time PCR

Primers were designed based on *CaBBX* gene sequences for real-time PCR by using the real-time PCR design tool in Integrated DNA Technologies (IDT, https://sg.idtdna.com/scitools/Applications/RealTimePCR/) (All primers are listed in Table S1). Real-time PCR application was carried out in a LightCycler® 480 Real-Time PCR Detection System (Roche, Hercules, Switzerland) with ChamQ SYBR Color qPCR Master Mix (Vazyme, Nanjing, China). The constitutive *actin* gene (Gen Bank accession No. AY572427) was used as an internal control and served as a standard gene for normalizing all mRNA expression levels [50]. A total of 20 μL reaction system contained 10 μL SYBR Color qPCR Master Mix, 1 μL cDNA samples, 0.4 μL of each primer (10 μM) and 8.2 μL ddH_2_O. The PCR thermal cycle conditions were as follows: denaturation at 95 °C for 30 s, 40 cycles of 95 °C for 10 s, and 58 °C for 20 s and 72 °C for 20 s. Fluorescence intensities were measured for qRT-PCR at the end of each cycle. A melting curve (61 cycles at 65 °C for 10 s) was performed directly to check for specific amplification. The relative gene expression was calculated by using the 2^-△△Ct^ method [51], the experiments were performed triplicate technological repeats. The SPSS statistics software (version 17.0) was used to analyze significant differences [52].

### Subcellular localization analysis

The subcellular localization of CaBBX proteins was predicted by Plant-mPLoc in Cell-PLoc 2.0 (http://www.csbio.sjtu.edu.cn/bioinf/plant-multi/) [53]. And four CaBBX proteins were chosen to verify the predication results. The four selected *CaBBX* genes were isolated from the cDNAs of pepper var. ‘Qingnong dried No.2’, and the amplified products were recombined into pMDC83 vector with green fluorescent protein (GFP), and then transferred to *Agrobacterium tumefaciens* Gv3101 strain for following infection. The onion inner epidermis was used for transforming with vectors with *CaBBX* genes. Fluorescence images were captured and analyzed using a Zeiss laser scanning confocal microscope TCS SP5 (Leica, Brunswick, Germany) and the LSM image software.

## Results

### Identification and characteristics of *BBX* genes in pepper

We searched PepperHub (Pepper Information Hub, http://pepperhub.hzau.edu.cn/) and PGP (Pepper Genome Platform, http://passport.pepper.snu.ac.kr/?t=PGENOME) with the conserved B-box domain HMM profile (PF00643) to obtain global putative *BBX* genes in pepper. Then the putative encoding protein sequences of these genes were further confirmed their B-box domain by using SMART, Pfam and InterProScan searches, six putative genes without B-box domain were removed. In total, we eventually identified 24 BBX genes in pepper, which were named *CaBBX1* to *CaBBX24*. Afterwards, the detailed information gene name, gene annotation ID, genomic position, gene length, theoretical isoelectric point, and molecular weight of their encoding protein were listed in Table 1.

**Table 1:** Information of the *BBX* gene family in pepper

Gene	Annotated CDS	Genomic position	Chr	CDS	AA	pIs	MV	Subcellular localization
CaBBX1	Capana02g003201	157,124,787–157,126,407	2	1224	407	5.27	43.89	Nucleus
CaBBX2	Capana02g003200	157,118,313–157,120,062	2	1197	398	5.32	45.39	Nucleus
CaBBX3	Capana02g003199	157,107,846–157,110,095	2	1215	404	5.41	44.56	Nucleus
CaBBX4	Capana01g004030	278,243,944–278,245,872	1	1032	343	5.45	37.93	Nucleus
CaBBX5	Capana12g000414	8,179,392–8,181,909	12	1071	356	5.29	39.43	Nucleus
CaBBX6	Capana07g000030	1,563,552–1,565,426	7	1155	384	6.56	42.54	Nucleus
CaBBX7	Capana00g004028	606,409,675–606,410,256	–	582	193	5.32	21.20	Nucleus
CaBBX8	Capana07g001114	154,056,816–154,057,654	7	693	230	5.03	24.83	Nucleus
CaBBX9	Capana00g004489	649,683,777–649,686,080	–	1281	426	5.07	46.87	Nucleus
CaBBX10	Capana03g003558	228,734,754–228,738,294	3	1164	387	5.45	43.34	Nucleus
CaBBX11	Capana00g001486	399,610,115–399,614,902	–	1410	469	6.75	51.67	Nucleus
CaBBX12	Capana11g002294	218,148,220–218,150,401	11	951	316	4.85	35.20	Nucleus
CaBBX13	Capana03g000377	5,273,465–5,275,489	3	1149	382	5.53	43.46	Nucleus
CaBBX14	Capana02g002620	148,180,402–148,183,481	2	624	207	8.47	22.97	Nucleus
CaBBX15	Capana08g002625	149,926,925–149,929,248	8	639	212	6.17	23.52	Nucleus
CaBBX16	Capana12g000659	18,213,299–18,215,385	12	960	319	6.50	35.22	Nucleus
CaBBX17	Capana04g000266	4,091,269–4,092,722	4	918	305	6.24	33.86	Nucleus
CaBBX18	Capana07g002062	212,563,126–212,570,473	7	894	297	4.98	32.01	Nucleus
CaBBX19	Capana09g000394	12,877,150–12,887,068	9	900	299	4.97	32.17	Nucleus
CaBBX20	Capana06g000735	11,697,317–11,699,646	6	702	233	5.00	25.98	Nucleus
CaBBX21	Capana08g002611	149,745,414–149,746,937	8	804	267	5.62	29.83	Nucleus
CaBBX22	Capana05g001195	84,622,805–84,628,926	5	1476	491	6.00	54.98	Nucleus
CaBBX23	Capana07g001588	191,773,542–191,774,577	7	744	247	4.60	27.40	Nucleus
CaBBX24	Capana00g004911	672,862,631–672,863,374	–	744	247	9.17	27.71	Nucleus

Note:Annotated CDS annotated coding DNA sequences, Genomic position, Chr chromosome, CDS coding DNA sequences, AA amino acid residues, pI theoretical isoelectric point, MV, Subcellular localization. The subcellular location results of pepper *BBX* genes were predicted by Plant-mPLoc in Cell-PLoc 2.0

These *BBX* genes showed diverse in length leads to the various length, theoretical isoelectric point, and molecular weight of their encoding protein. These *BBX* genes with sequence of 582 to 1476 bp encoded ranging from 193 (least, *CaBBX7*) to 491 (most, *CaBBX22*) amino acid residues. And the isoelectric points of 24 BBX proteins were ranging from 4.60 (lowest, *CaBBX23*) to 9.17 (highest, *CaBBX24*), with the molecular weights of 21.20 ~ 54.98 kDa (Table 1).

### Phylogenetic analysis of the CaBBX family

**Fig. 1:**
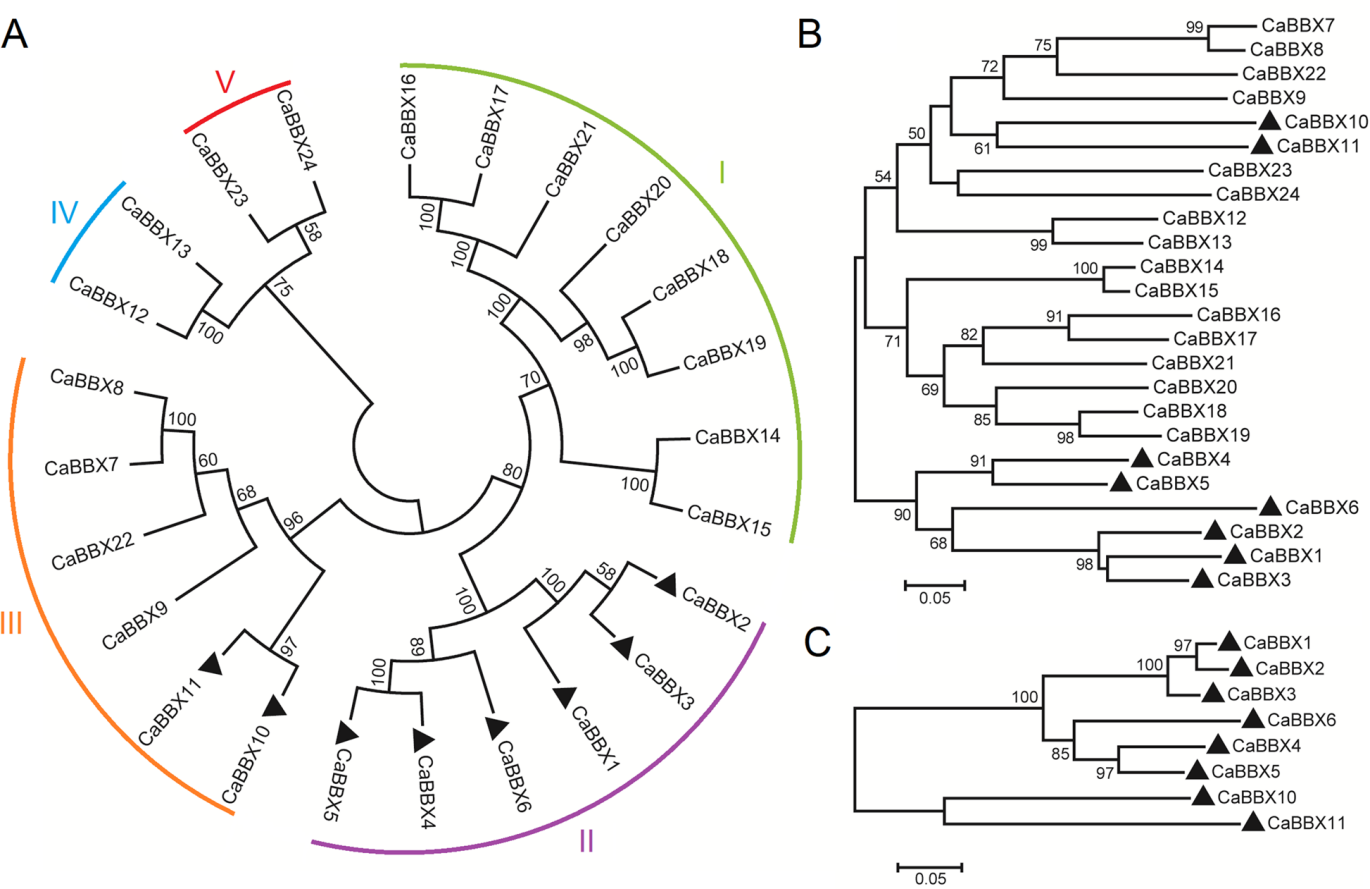
Phylogenetic analysis of *BBX* genes in pepper. **A**. Phylogenetic tree of *BBX* members from pepper. **B**. The trees shown were based on the alignments of the first B-box domain sequences. **C**. The trees shown were based on the alignments of the protein sequences with the two B-box plus the CCT domain. Ca: *Capsicum annuum* L. The protein sequences used to construct the tree were CaBBX1 (Capana02g003201), CaBBX2 (Capana02g003200), CaBBX3 (Capana02g003199), CaBBX4 (Capana01g004030), CaBBX5 (Capana12g000414), CaBBX6 (Capana07g000030), CaBBX7 (Capana00g004028), CaBBX8 (Capana07g001114), CaBBX9 (Capana00g004489), CaBBX10 (Capana03g003558), CaBBX11 (Capana00g001486), CaBBX12 (Capana11g002294), CaBBX13 (Capana03g000377), CaBBX14 (Capana02g002620), CaBBX15 (Capana08g002625), CaBBX16 (Capana12g000659), CaBBX17 (Capana04g000266), CaBBX18 (Capana07g002062), CaBBX19 (Capana09g000394), CaBBX20 (Capana06g000735), CaBBX21 (Capana08g002611), CaBBX22 (Capana05g001195), CaBBX23 (Capana07g001588), CaBBX24 (Capana00g004911). The phylogenetic tree was constructed based on peptide sequences using the Neighbor-Joining method

To identify the phylogenetic relationship and division of CaBBX proteins, we constructed the phylogenetic tree of BBX family proteins in pepper (Fig. 1). The phylogenetic analysis of the CaBBXs with AtBBXs, PtBBXs, OsBBXs and SlBBXs was also carried out to confirm the subfamily clustering of CaBBXs (Fig. S1). The division of 24 CaBBXs were not even on the phylogenetic tree (Fig. 1A). All the 24 CaBBXs were divided into five subfamilies with the similarity of the amino acid sequences based on previous studies in tomato [1]. In addition, the phylogenetic relationship of first B-box domain was constructed, as well as two B-box and one CCT domain (Fig. 1B and C). In total, there were eight CaBBXs classified into subclass I, whose contain two B-box domains, making up the largest subclass. The subclass II and III both contained six members, and only two members (CaBBX12 and 13) clustered together in subclass IV, and CaBBX23 and 24 aligned together in subclass V. Members from subclass II owed two B-box domains and one CCT domain, while only one BBX proteins (CaBBX13) possessed one B-box and one CCT domain belonging to subclass IV. Other CaBBX proteins only contained one or two B-box domains without CCT domain. Moreover, based on the phylogeny of BBXs in *Arabidopsis*, rice, tomato and *Populus tomentosa*, most of the BBXs with two B-box domains and one CCT domain were classified into subgroup II, and most of whom with two B-box domains and none CCT domain were classified into subgroup I. While, BBXs contain one single B-box domain were most together classified into subgroup V.

### Domains, motif structure and gene structure analysis

**Fig. 2:**
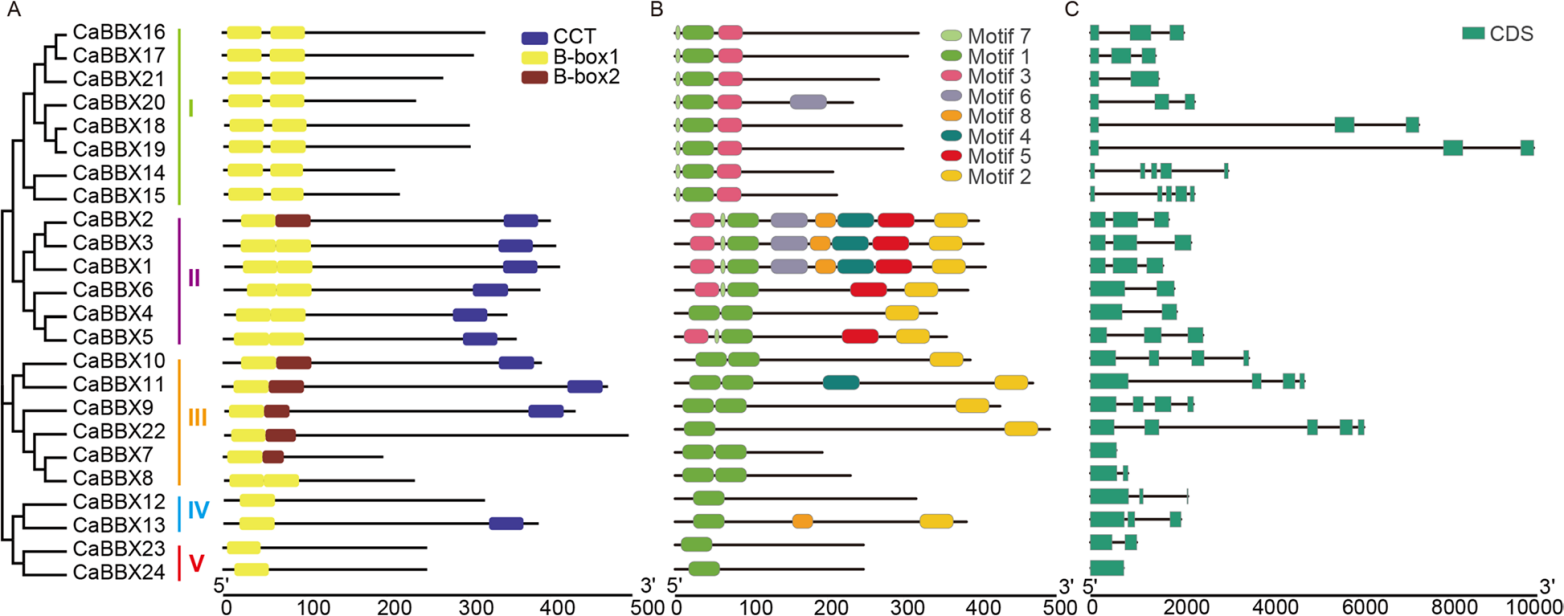
The domain, conserved motifs and gene structures of the BBX family members in pepper. **A**. The domain of BBX family members in pepper. **B**. The distribution of conserved motifs of BBX family members in pepper. **C**. The gene structures of BBX family members in pepper. The boxes and lines denote exons and introns, respectively. Eight conserved motifs of each subfamily were displayed in different colors. The scale on the bottom is in base pair (bp)

To determine the domains, motif structure and gene structure of CaBBXs, the conserved domain information were confirmed by CDD in NCBI, and motif and CDS were also plotted to identify structure analysis of CaBBXs (Fig. 2).

Eight motifs were identified in these CaBBXs, members from same subclass shared similar motifs according to the phylogenetic relationship (Fig. 2A). For example, all the members from subclass I contained motif 1, 3 and 7, only CaBBX20 also owed another motif 6. While, except CaBBX4 only owed two motif 1 and one motif 2, CaBBXs from subclass II possessed maximum motifs, containing motif 1, 2, 3, 5 and 7; moreover, three of them (CaBBX1, 2 and 3) contained all the 8 identified motifs. Other members carried one or two motif 1, several of them contained motif 2; in addition, CaBBX11 and 13 also had a motif 4 and 8 located in middle of their amino acid sequence, respectively. The detail sequence information of these eight motifs were shown in Fig. S2.

Furthermore, the gene structures of *CaBBXs* were constructed with TBtools by gff file from pepper genome 2.0 [49]. Among 24 *CaBBXs*, only *CaBBX24* had no intron, others had one to five exons. To make clear the domains arrangement, we also plotted domains on the CDS directly. Nine BBX proteins were identified containing two B-box and a CCT domain, five of them share two same B-box, and four possessed two different B-box domains. Only one BBX proteins (CaBBX13) possessed one B-box and one CCT domain, while three and eleven CaBBX proteins contained one and two B-box without CCT domain, respectively (Fig. 2B). These results were consistent with the phylogenetic divergence analysis. Except for subclass I members, the B-box domains of members from other subclasses were located in the beginning of first exon. The B-box domains of subclass I members were on the first three exon. CCT domain were situated in the terminal of last two exon. Moreover, the two B-box (B-box1 and B-box2 domains) share similar conserved sequences and Zinc finger domain.

### Chromosomal localization and duplication of *BBX* genes in pepper

**Fig. 3:**
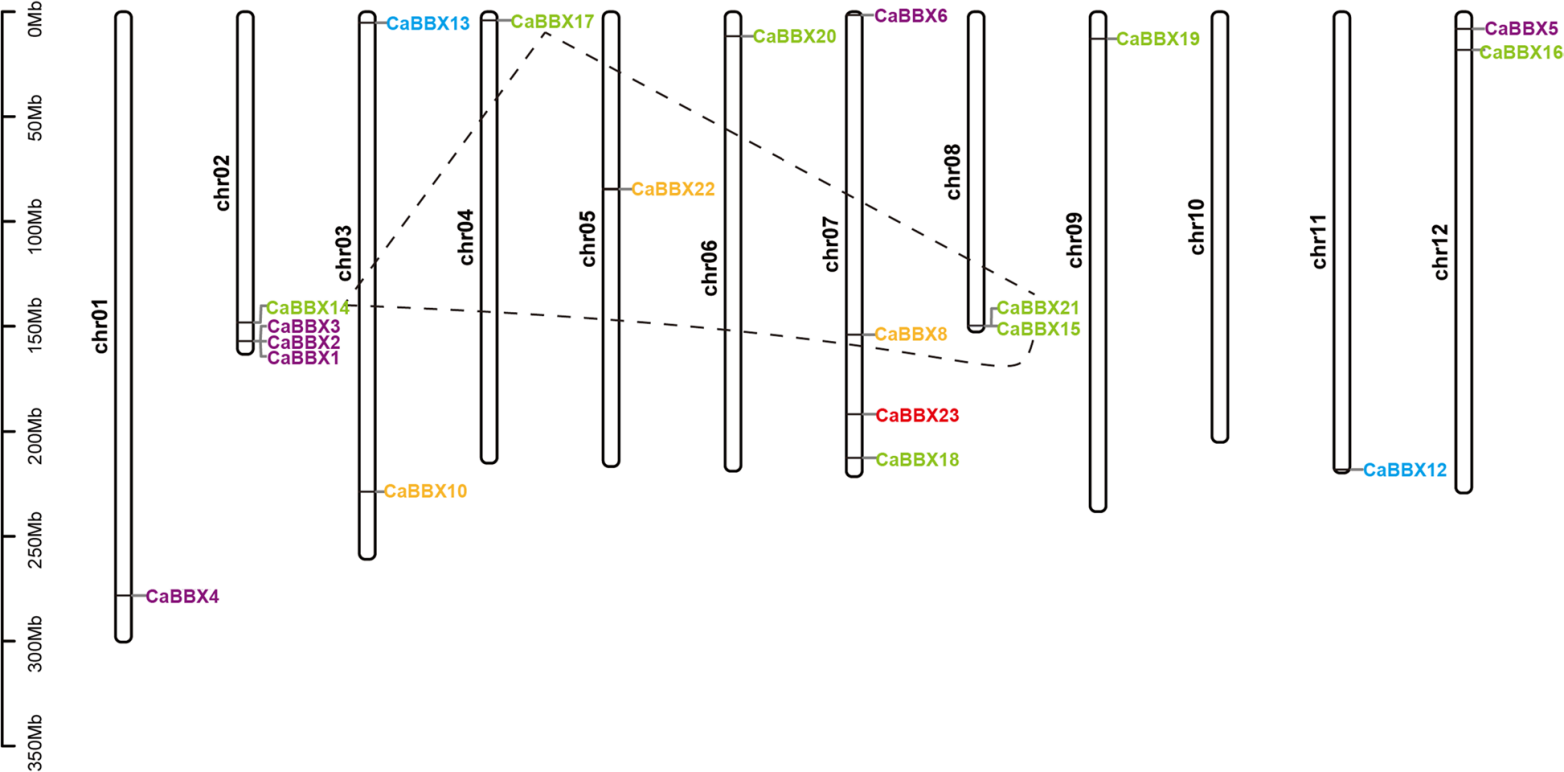
Chromosome distribution and segmental duplication of pepper *BBX* genes. Chromosomal mapping was based on the physical position in 12 pepper chromosomes. The scale on the left is in megabases (Mb). Different color represented the classification of *CaBBX* genes, green: subclass I; purple: subclass II; orange: subclass III; blue: subclass IV; red: subclass V. The chromosome numbers are indicated at the side of each bar. The segmental duplicated genes are connected by lines

We have plotted the *CaBBX* genes to the chromosomes of pepper genome to confirm their genomic distribution (Fig. 3). Except for four *CaBBX* genes (*CaBBX7*, *CaBBX9*, *CaBBX11* and *CaBBX24*), 20 *CaBBX* genes were distributed unevenly on 11 of 12 pepper chromosomes, no gene was on chromosome 10. Both chromosome 02 and 07 possessed four *CaBBX* genes, making up the maximum number of genes among all these 12 chromosomes. In addition, only one *CaBBX* gene was located on chromosome 01, 04, 05, 06, 09 and 11, respectively; and two on chromosome 03, 08 and 12, respectively.

**Fig. 4:**
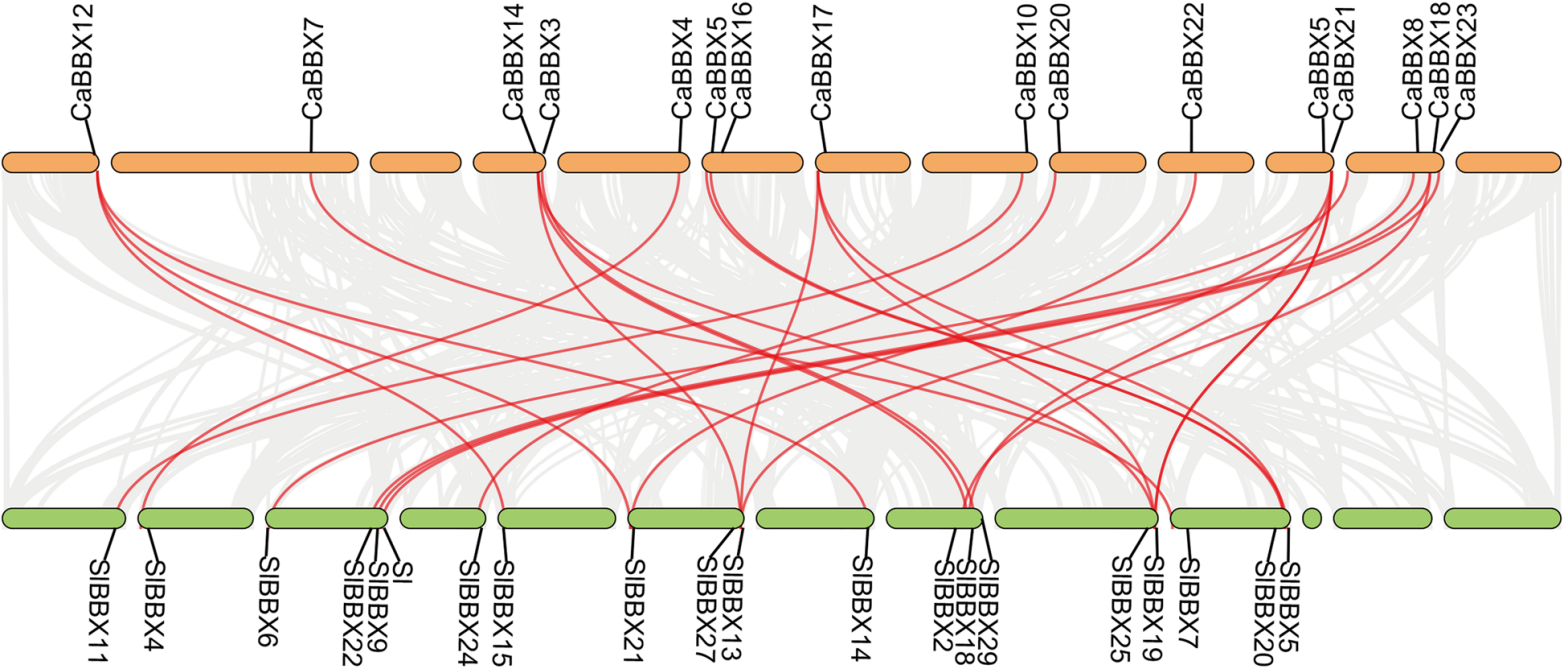
Chromosome distribution and duplication events of pepper and tomato BBX genes. The segmental duplicated genes are indic*ated in red an*d connected by lines. (Orange pillar represent *Capsicum annuum L* chromosome, from left to right: chr11, chr00, chr10, chr02, chr01, chr12, chr04, chr03, chr06, chr05, chr08, chr07, chr09; Green pillar represent *Lycopersicon esculentum* chromosome,from left to right: chr09, chr08, chr07,chr06, chr05, chr04, chr03, chr02, chr01, chr12, chr00, chr11, chr10)

Potential duplication within pepper were marked on the 12 chromosomes by using TBtools [49]. Expect *CaBBX7* (duplicated with *CaBBX8*) was not located on pepper chromosomes, the other three duplications only occurred on the 3 of 12 chromosomes, and these duplicated genes (CaBBX14, CaBBX15, CaBBX17 and CaBBX21) were all belonged to subgroup I (Fig. 3). And all the duplication events occurred between two different chromosomes, not within the same chromosome. In addition, we constructed a collinearity relationship analysis to identify the duplication events of BBX genes between pepper and the model Solanaceae plant tomato (Fig. 4). Twenty-six pairs of BBX genes were identified duplicate between pepper genome and tomato genome. All the subgroups of *BBX* genes involved in duplication. Among them, 13 pairs of subgroup I members play part in the replication events, account for half of the total duplication events. And we found 4 pairs of subgroup II and III members, 3 pairs of subgroup IV members and 2 pairs of subgroup V members were homologous in the pepper and tomato genome (Fig. 4).

### Organ development expression analysis of *BBX* genes in pepper

**Fig. 5:**
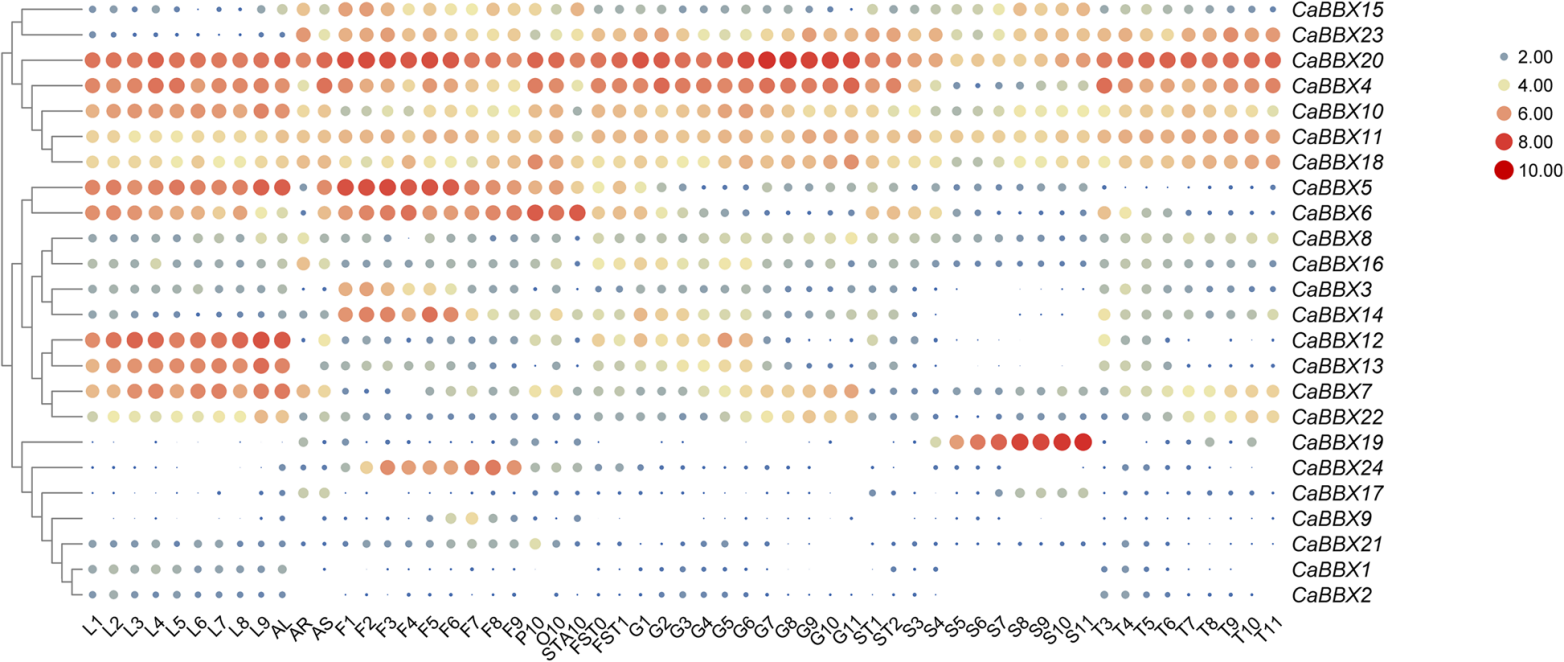
Expression profiles of the pepper *CaBBX* genes in different organs, tissues and developmental stages. Data were normalized based on the mean transcript levels (log2scale) of each gene in all tissues analyzed. Genes were hierarchically clustered based on average Pearson’s distance metric and ‘average linkage’ method. Red and green circular indicate high and low expression levels, respectively, for each gene. 2 d (L1), 5 d (L2), 10 d, 15 d (L4), 20 d (L5), 25 D (L6), 30 d (L7), 40 d (L8), 50d (L9), 60 d (L10) after the emergence of new leaves. Leaf (AL), root (AR), stem (AS). According to the size of flowers, the flowers of different development stages can be divided into nine stages (F1, F2, F3, F4, F5, F6, F7, F8, F9) such as young bud stage and white stage, and the flowers that open on the same day (F10). Ovary (O10) and anther (STA10). The fruit pollination three days (FST0), seven days (FST1), from the third stage (10d after pollination), the fruit was divided into three tissues: pulp (G), placenta (T) and seed (S). 10 days (G1), 15 days (G2), 20 days (G3), 25 days (G4), 30 days (G5), 35 days (G6), 40 days (G7), 45 days (G8), 50 days (G9), 55 days (G10), 60 days (G11) after pollination

To investigate the tissue-specific and developmental expression pattern of all the *CaBBX* genes, we performed the heatmap by using TBtools based on the transcript data from Pepper Information Hub (http://pepperhub.hzau.edu.cn/) [49]. Several CaBBX genes showed organ-specific expression pattern, such as CaBBX19, expressed specifically in seed, respectively, and expressed arise as the tissues’ development (Fig. 5). This result indicated that CaBBX19 may play an important role in seed morphogenesis development, respectively. *CaBBX7*, *CaBBX12*, *CaBBX13* and *CaBBX22* mainly expressed in leaf, may showed their regulatory function in pepper leaf (Fig. 5). Additionally, CaBBX5 and CaBBX6 showed high expression levels in leaf and flower, CaBBX3 and CaBBX14 specifically expressed in the early developmental stage of flower, CaBBX4 and CaBBX20 expressed in almost all the detected tissues, and expressed most highly in fruit development, especially in the pericarp, however, expect in the seed (Fig. 4). This result may indicate that *CaBBX4* and *CaBBX20* involved in the pericarp development (such as pigmentation, enlargement, and so on).

**Fig. 6:**
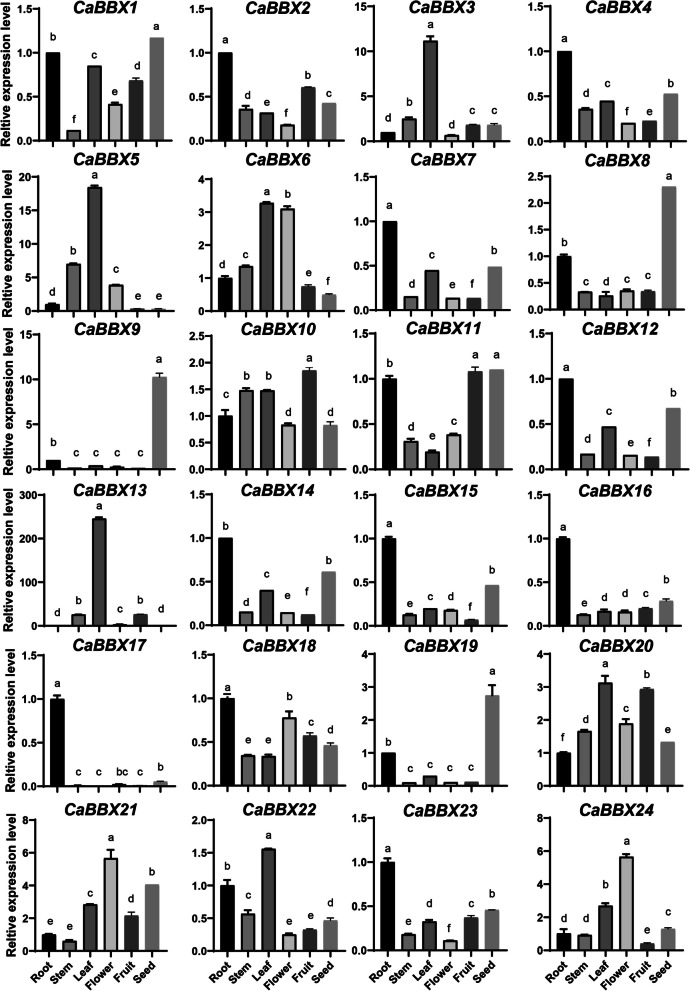
The expression of *CaBBXs* in different pepper tissues. Different tissues were arranged as ‘root, stem, leaf, flower, fruit and seed’. Three independent biological experiments were performed (*P* < 0.05)

Furthermore, we also investigated the expression levels of 24 *CaBBX* genes by qRT-PCR. In particularly, *CaBBX19* showed the highest expression level in seed, and *CaBBX24* was expressed more highly in flower than that in the other tissues, indicating that *CaBBX19* and *CaBBX24* play essential roles in controlling pepper seed development and flowering (Fig. 6). *CaBBX13* and *22* showed highly expression in leaf, while *CaBBX6* expressed highly in both leaf and flower. These results were consisting with that of RNA-seq data transcript levels.

### Expression analysis of *BBX* genes under abiotic stress in pepper

**Fig. 7:**
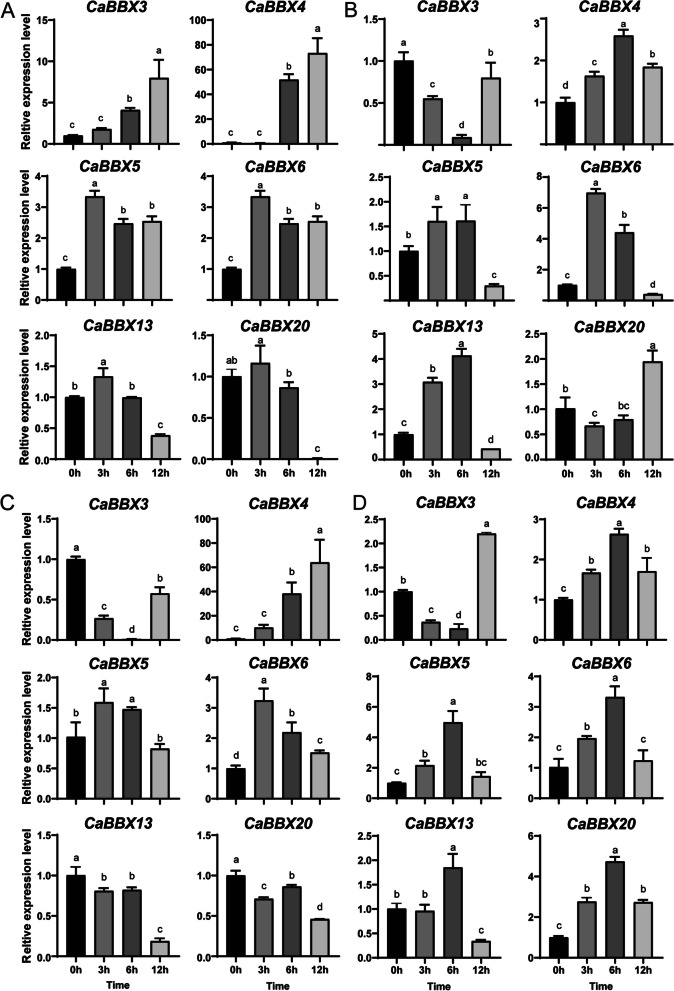
The expression of *CaBBXs* under different abiotic stresses. **A**. qRT-PCR transcript analysis of 6 selected *CaBBX* genes under cold stress. **B**. qRT-PCR transcript analysis of 6 selected *CaBBX* genes under heat stress. **C**. qRT-PCR transcript analysis of 6 selected *CaBBX* genes under drought stress. **D**. qRT-PCR transcript analysis of 6 selected *CaBBX* genes under salt stress. Three independent biological experiments were performed (P < 0.05)

The expression levels of *CaBBX* genes under cold, heat, salt and drought stress were investigated by qRT-PCR analysis, to analyze *CaBBX* genes in response to abiotic stress (Fig. 7). We selected six *CaBBX* genes (*CaBBX3*, *CaBBX4*, *CaBBX5*, *CaBBX6*, *CaBBX13* and *CaBBX20*) potentially responding to abiotic stress base on transcriptome data of different stress treatment from the Pepper Genome Database (Fig. S3, http://pepperhub.hzau.edu.cn/). The expression levels were detected under high temperature, low temperature, NaCl and PEG6000 treatment at 3 h, 6 h, 12 h. In low temperature treatment, four of these six *CaBBX* genes showed up-regulated expression, except for *CaBBX13* and *CaBBX20*, they both showed a high expression level under 3 h cold treatment, but rapidly down-regulated afterwards 6 h (Fig. 7A). Under heat stress condition, the expression of *CaBBX4*, *CaBBX5*, *CaBBX6* and *CaBBX13* got a peak at 3 or 6 h treatment, and then decreased at 12 h treatment, while *CaBBX3* and *CaBBX20* exhibited the opposite expression pattern (Fig. 7B). There were three *CaBBX* genes (*CaBBX4*, *CaBBX5* and *CaBBX6*) expressed up-regulated under drought stress, and the expression levels of other three genes were decreased (Fig. 7C). Only the expression of *CaBBX3* were repressed under salt condition, and showed an early down-regulation at 3 and 6 h, but rapidly increased its expression even more so than the control at 12 h, while *CaBBX4*, *CaBBX5*, *CaBBX6* and *CaBBX20* were expressed more highly than control. Additionally, *CaBBX13* showed an up-regulation at 6 h, but rapidly decreased with a more lower expression level than control at 12 h treatment (Fig. 7D). These results indicated that the six *CaBBX* genes may involve in responding to abiotic stress.

### Expression analysis of Ca*BBX* genes in response to exogenous hormones

**Fig. 8:**
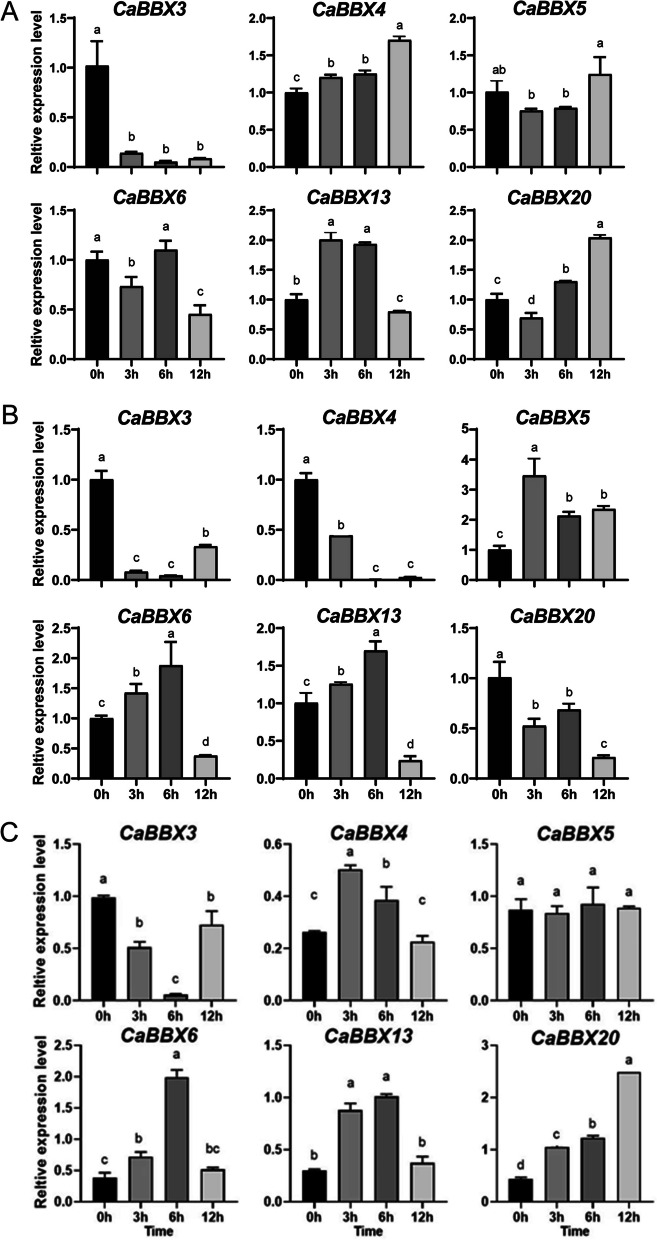
The expression of *CaBBXs* under different hormone treatment. **A**. qRT-PCR transcript analysis of 6 selected *CaBBX* genes under ABA treatment. **B**. qRT-PCR transcript analysis of 6 selected *CaBBX* genes under MeJA treatment. **C**. qRT-PCR transcript analysis of 6 selected *CaBBX* genes under SA treatment. Three independent biological experiments were performed (P < 0.05)

In addition, the expression pattern of *CaBBX* genes under ABA, MeJA and SA treatment were also measured because of their important part in plant growth, development and in response to biotic and abiotic stress. The expression profiles of six *CaBBX* genes potentially involved in response to abiotic stress were also investigated under ABA, MeJA and SA treatment at 3 h, 6 h, 12 h (Fig. 8). Three of these detected genes (*CaBBX4*, *CaBBX13* and *CaBBX20*) were induced to express more highly by ABA treatment than control at different time-point treatment. *CaBBX4* was up-regulated during the entire treatment. However, the expression level of *CaBBX13* was increased significantly at 3 and 6 h treatment, but was repressed at 12 h treatment. And the expression of *CaBBX3* was repressed by ABA significantly at all the three time-point (3, 6 and 12 h) treatment. *CaBBX5* showed no obviously significant expression levels, and the expression of *CaBBX6* at 3 and 12 h treatment was lower than that of control (Fig. 8A). The expression of *CaBBX3*, *CaBBX4* and *CaBBX20* were repressed dramatically after MeJA application, while the expression of other three genes (*CaBBX5*, *CaBBX6* and *CaBBX13*) were up-regulated at early (3 h and/or 6 h) treatment stage, but *CaBBX6* and *CaBBX13* were expressed decreased rapidly at 12 h treatment (Fig. 8B). Except for *CaBBX3* and *CaBBX13*, all the other four selected *CaBBX* genes were induced to expressed at a higher level by exogenous SA, and reached peak at 6 h treatment. Except for *CaBBX3* and *CaBBX5*, all the other four selected *CaBBX* genes were induced to expressed at a higher level by exogenous SA at different treatment stages (Fig. 8C).

### Subcellular localization of CaBBXs

**Fig. 9:**
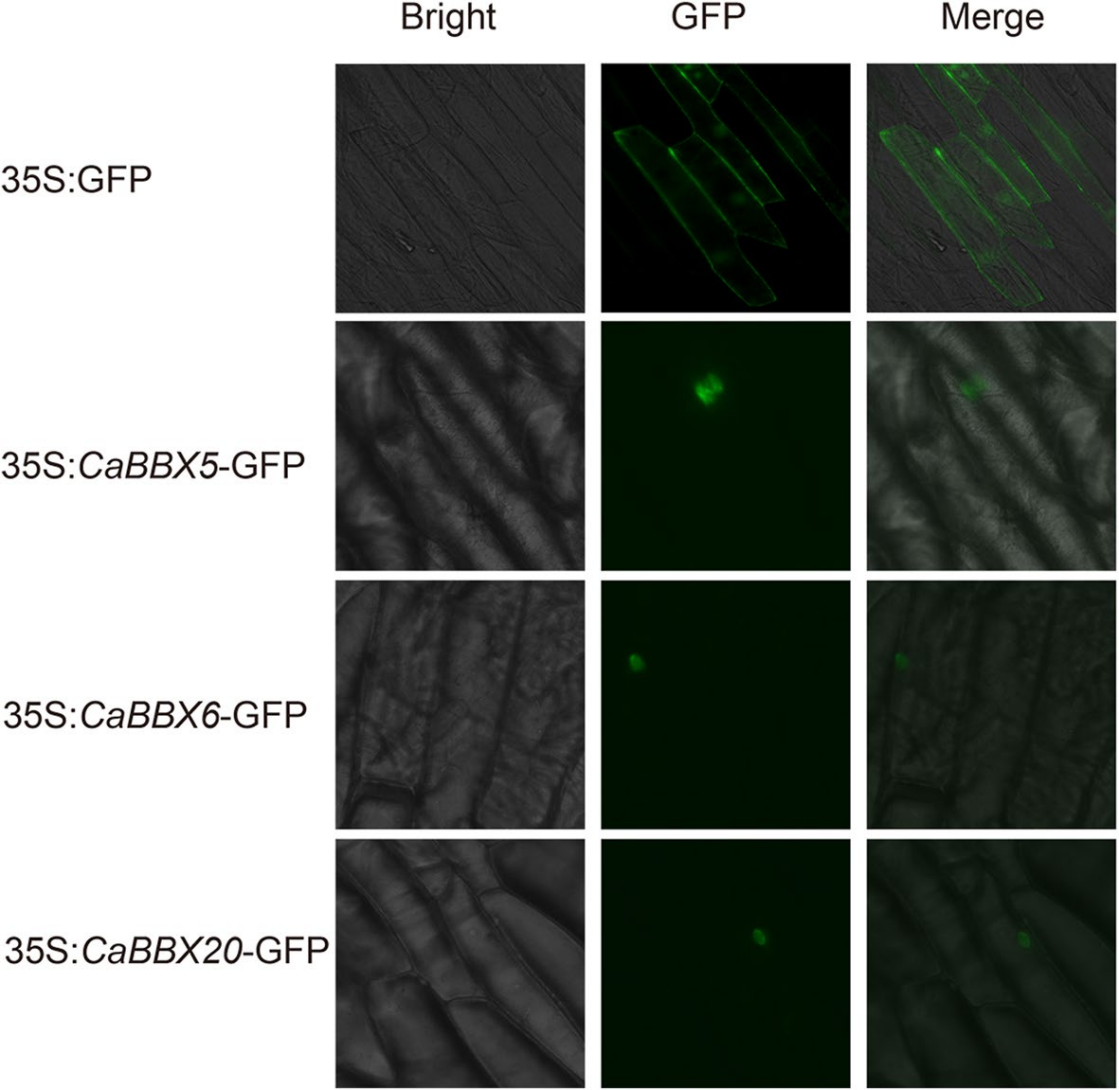
Subcellular localization of three GFP-fused CaBBX proteins. The three CaBBX-GFP fusion proteins (CaBBX5-GFP, CaBBX6-GFP and CaBBX20-GFP) were transiently expressed in inner epidermis of onion and observed by fluorescence microscopy 24 h later

The subcellular localization of proteins was analyzed to further understand the function. We first predicted the subcellular localization by Plant-mPLoc in Cell-PLoc 2.0 (Table 1). All of the 24 CaBBXs were identified to be located in nucleus with the highest possibility. We selected three *CaBBXs* (*CaBBX5*, *6*, and *20*) which were strongly induced or repressed by abiotic stress, hormones or showed organ-specific and organ developmental expression patterns for a transient expression assay using GFP-fused BBX proteins with onion epidermis. All the three CaBBXs were found to be in the nucleus (Fig. 9). The result of CaBBX20, CaBBX5 and 6 was consistent with the most preferentially predication.

## Discussion

### Genome evolution of the B-box transcription factors in pepper

BBX transcription factors were widely identified in many higher plants, such as *Arabidopsis* [2], rice [11], tomato [10], apple [13], pear [12], and so on [14, 42, 54]. The CaBBX was characterized the structure, phylogenetic relationship, chromosomal location, expression patterns and subcellular localization in our study. Based on the sequence similarity, the BBXs can be classified into five different subclasses [25]. The *BBX* genes were investigated in 13 different high plants, and the total number among these plant species is relative stable with about 30 members (Fig. S4), expect for several species which experienced whole genome duplication. The triplication events occurred in Chinese cabbage genome since its divergence from *Arabidopsis*, resulting in the genome size of Chinese cabbage (485 Mb) more than three times larger than that of *Arabidopsis* (125 Mb) [55]. However, there were 75 BBX members in Chinese cabbage, only twice of that in *Arabidopsis* (32 members). Moreover, the number of GmBBXs (50 members) is more than three times of *Medicago truncatula* (15 members), soybean has undergone a specific tetraploidy [56]. The gene number is less than the genome duplication ploidy may result from alterative actions during the evolution. The number of *BBX* genes in the three Solanaceae species (tomato, pepper, and potato) is relatively stable. However, the number of each subfamily among these plants differed a lot, this may indicate that the function of BBX belonging to different subclasses varied resulting from the differentiation of B-box proteins.

In addition, the diversity of are motif and intron-exon structure important for the evolution of many gene families. In subclass I, except for CaBBX20, all the other six CaBBXs had six same motifs (motif 1, 3 and 7). And CaBBX1–3 in a clustering clade within subclass II, contained all the same motif structure (Fig. 2A). It was identified that the CaBBXs in same phylogenetic clade shared the similar motif structures resulting in a conserved pattern in the evolution of CaBBX transcription factors.

Moreover, we found that the intron-exon structure of CaBBXs in same subclass also differed a little between the diverse clades, but are highly similar within the same phylogenetic clade. Thus, it is also indicated that the evolution of CaBBXs were relatively conserved with low occurrence of mutation events. However, the full genomic sequences of most *CaBBX* genes were below 5 kb, but not for that of *CaBBX18*, *19* and *22* that were 6 kb, 7 kb and 10 kb, respectively (Fig. 2B). Therefore, mutation events might still exist in highly conserved genes resulting from the evolutional diversity.

### Gene duplication of BBX genes in pepper

As is known, genetic novelty mainly caused by gene or genome duplication events, and gene family expansion was primarily resulting from gene duplication [57–59]. Novel BBX genes arise through divergence of duplicate genes after either single gene duplication, segmental duplication, or whole-genome duplication [57, 60]. The chromosomal location of *CaBBX* genes indicated that the distribution of BBX genes in pepper genome is not even, this result may cause by genome duplication which occurred throughout plant evolution [61]. Three pairs of potential duplicate *BBX* genes were found in pepper chromosomes, and a single *CaBBX* gene, *CaBBX7*, was located on ChrUM. Interestingly, all the three pairs of duplicate genes belonged to the same subgroup I, and *CaBBX14* duplicated with *CaBBX15* and *CaBBX17*. While *CaBBX17* duplicated with *CaBBX21*, which was clustered with *CaBBX15* located on chromosome 08 (Fig. 3). In addition, 26 *CaBBX*-*SlBBX* gene pairs were identified by collinearity analysis. Among them, 13 subgroup I gene pairs were found counted for the largest number of replication events. These results suggest that the expansion of CaBBX genes in pepper chromosome is affected by the network and duplication events of pepper.

The evolution of CaBBX transcription factors might indicate that their diverse function in tolerance to abiotic and biotic stress, responding to phytohormone, and even in plant growth and developmental processes, such as seedling photomorphogenesis, shade avoidance, photoperiodic regulation of flowering [25]. And the specific function of CaBBX transcription factors involved in plant development and stress tolerance were still looking forward to be elucidated.

### Tissue-specific and developmental expression patterns of *CaBBX* genes

In several model plant, Arabidopsis, rice or tomato, the BBXs participated in seedling photomorphogenesis, such as flowering, hypocotyl growth, pigmentation and cotyledon unfolding [8, 27, 33, 62, 63]. Here, the transcript expression levels of 24 *CaBBXs* were investigated in ten organs or tissues, as well as during leaf, flower, pulp, placenta and seed development (Fig. 5). Over-expression of *CONSTANS-LIKE 5* can induce flowering under short-day condition in *Arabidopsis* [28]. AtBBX30 and AtBBX31 were negatively regulated by HY5, which directly binding to the G-box cis-element present in their promoters, negatively regulate photomorphogenesis in *Arabidopsis* [22]. While the homology of *AtBBX30* and *AtBBX31*, *CaBBX24* was expressed at a high level in flower (Figs. 5 and 6). AtBBX4 is a key component involved in the phyB (Phytochrome B)-PIF3 (PHYTOCHROME INTERACTING FACTOR 3) regulatory module to promote photomorphogenesis [23]. *CaBBX4* clustered with *AtBBX4*, expressed highly in flower and pulp, while the flower and fruit development were related to photomorphogenesis. *OsCO3*, a *BBX* gene in rice, can regulating flowering time by repressing the expression of FT-like genes under SD conditions [30]. Recent study suggested that AtBBX28 negatively regulates photomorphogenesis by repressing HY5 activity [21], the homologous *BBX* in pepper is *CaBBX23*, showed lower expression in flower in RNA-seq data transcript levels and qRT-PCR analysis (Figs. 5 and 6), with the similar function of AtBBX28. *CaBBX7*, *12* and *13* were expressed a relatively high level in the early stage of leaf development, may suggest they involved in cotyledon unfolding. In addition, *CaBBX5* and *6* were also expressed at a high level in both leaf and flower, suggesting they were involved in photomorphogenesis. *CaBBX19* expressed only in seed, and had a gradually increasing expression pattern during seed development, indicating that its important roles in seed formation and growth. Besides, AtBBX24 and AtBBX25 were identified interacting with HYH, an HY5 HOMOLOG, to regulate Arabidopsis seedling development [64], AtBBX24 and AtBBX25 were clustering with CaBBX20 by our phylogenetic analysis (Fig. S1). Recently, a tomato BBX transcription factor, SlBBX20 modulates fruit pigmentation by directly activating the rate-limited enzyme of carotenoid biosynthesis *PSY1* [62]. *CaBBX20* was classified into group I homologous with AtBBX24 and AtBBX25, and expressed a relatively high level in pepper pulp (Fig. 4), this may indicate that CaBBX20 evolved special function in fruit development.

### Stress and hormones induced expression of *BBX* genes in pepper

BBX transcription factors were also proved to be involved in response to stress and phytohormones [27, 35]. AtBBX18 was detected to be a negative regulator in heat tolerance in *Arabidopsis* [39]. In pepper, we found the expression profiles of *CaBBX4*, *5*, *6*, *13* and *20* were significantly similar to be induced high expression by heat and salt stress. Moreover, *CaBBX20* homologous with *AtBBX24*, was induced by salt stress (Fig. 7D), AtBBX24 also called STO, was identified to be a salt tolerance factor, which can enhance *Arabidopsis* root growth under salt stress treatment [35]. And it was found that *SlBBX20* was up-regulated in M82 (cultivated tomato M82 is sensitive to stress) under drought stress in tomato [65]. Similarly, *CaBBX4* was dramatically up-regulated (up to 10 ~ 60 fold comparing with control) under drought stress, may indicated that they shared the similar function in responding to drought stress.

Phytohormones are important for plant growth, development and also involved in tolerance to biotic and abiotic stress. Recently, an apple B-box protein BBX37 was identified that regulates jasmonic acid mediated cold tolerance through the JAZ-BBX37-ICE1-CBF pathway [41], MdBBX10 significantly enhanced abiotic stresses tolerance by ABA signalling [66].BBX19 belonged to subgroup IV, interacts with ABF3 to affect drought tolerance negatively in chrysanthemum [67]. All these selected *BBX* genes were found to be responded to ABA, MeJA and SA. Except for *CaBBX3*, other five *CaBBX* genes were up-regulated expressed by ABA and SA application. This result may indicate that *CaBBX4*, *5*, *6*, *13* and *20* as positive factors response to ABA and SA signaling involved in pepper plant growth or biotic and abiotic stress tolerance. While, under MeJA condition, only *CaBBX5* showed up-regulated expression at all three treated stages, others were down-regulated at different degrees, especially *CaBBX3* and *4*. This result may indicate that *CaBBX3*, *4*, *6*, *13* and *20* as negative factors response to later stage of MeJA signaling.

## Conclusion

In this study, we carried out a genome-wide analysis of 24 *CaBBX* genes, the phylogenetic analysis, domain, motif & gene structure, gene chromosome location, were performed preliminarily. In addition, several *CaBBX* genes were induced by abiotic stress and exogenous phytohormones, some expressed tissue-specific and variously at different developmental stage. And subcellular localization experiment was also investigated to further understand the potential function of *CaBBX* genes, it was indicated that they act as nucleus-localized transcription factors. Overall, out data might be a foundation in the identification of *CaBBX* genes, and a further understanding of their biological function in future studies.

### Supplementary Information

**Additional file 1: Table S1**. Primer sequences used for real-time qRT-PCR amplification.

**Additional file 2: Figure S1**. Phylogenetic tree of BBX members from *Capsicum annuum*, *Solanum lycopersicum*, *Arabidopsis thaliana*, *Oryza sativa * and *Populus trichocarpa* (Ca represent *Capsicum annuum*; Sl represent *Solanum lycopersicum*; At represent *Arabidopsis thaliana*; Os represent *Oryza sativa*; Pt represent *Populus trichocarpa*). The members marked in circle contain two B-BOX and one CCT domains, and in triangle contain two B-BOX domains (red represent *Capsicum annuum*; green represent *Solanum lycopersicum*; black represent *Arabidopsis thaliana*; purple represent *Oryza sativa*; blue represent *Populus trichocarpa*).

**Additional file 3: Figure S2**. Amino acid arrangement order of motif of BBX family.

**Additional file 4: Figure S3**. Expression profiles of *CaBBX* genes under different abiotic stresses. The first character C, F, H and N of the abbreviation on the bottom represent control, freezing, heat and NaCl treatment, respectively; the second character L and R represent leaf and root, respectively; the number 0, 0.1 and 0.2 represent 0h treatment; and the number 1, 2, 3, 4, 5 and 6 represent 1h, 1.5h, 3h, 6h, 12h and 24h treatment.

**Additional file 5: Figure S4**. The classification of *BBX* genes investigated in 13 higher plants (A-E representative five different subclasses in the BBXs Groups I ~ V, respectively).

## Data Availability

All data generated or analyzed during this study are included in this article (and its supplementary information files) or are available from the corresponding author on reasonable request. Sequence data from this article can be found in Arabidopsis Information Resource (https://www.arabidopsis.org/) under the following accession numbers: AtBBX1 (AT5G15840), AtBBX2 (AT5G15850), AtBBX3 (AT3G02380), AtBBX4 (AT2G24790), AtBBX5 (AT5G24930), AtBBX6 (AT5G57660), AtBBX7 (AT3G07650), AtBBX8 (AT5G48250), AtBBX9 (AT4G15250), AtBBX10 (AT3G21880), AtBBX11 (AT2G47890), AtBBX12 (AT2G33500), AtBBX13 (AT1G28050), AtBBX14 (AT1G68520), AtBBX15 (AT1G25440), AtBBX16 (AT1G73870), AtBBX17 (AT1G49130), AtBBX18 (AT2G21320), AtBBX19 (AT4G38960), AtBBX20 (AT4G39070), AtBBX21 (AT1G75540), AtBBX22 (AT1G78600), AtBBX23 (AT4G10240), AtBBX24 (AT1G06040), AtBBX25 (AT2G31380), AtBBX26 (AT1G60250), AtBBX27 (AT1G68190), AtBBX28 (AT4G27310), AtBBX29 (AT5G54470), AtBBX30 (AT4G15248), AtBBX31 (AT3G21890), AtBBX32 (AT3G21150). The sequences of VvBBX1 (VIT_214s0083g00640.2), VvBBX2 (VIT_211s0052g01800.1), VvBBX3 (VIT_204s0008g07340.1), VvBBX4 (VIT_212s0057g01350.2), VvBBX5 (VIT_200s0194g00070.1), VvBBX6 (VIT_207s0104g01360.1), VvBBX7 (VIT_201s0146g00360.1), VvBBX8 (VIT_214s0068g01380.1), VvBBX9 (VIT_201s0011g04240.1), VvBBX10 (VIT_219s0014g05120.1), VvBBX11 (VIT_212s0059g02500.1), VvBBX12 (VIT_201s0011g03520.1), VvBBX13 (VIT_203s0038g00690.1), VvBBX14 (VIT_204s0023g03030.1), VvBBX15 (VIT_218s0001g13520.1), VvBBX16 (VIT_203s0038g00340.1), VvBBX17 (VIT_218s0089g01280.1), VvBBX18 (VIT_219s0014g00350.1), VvBBX19 (VIT_205s0102g00750.1), VvBBX20 (VIT_219s0014g03960.1), VvBBX21 (VIT_212s0134g00400.1), VvBBX22 (VIT_212s0059g02510.1), VvBBX23 (VIT_200s0203g00210.1), VvBBX24 (VIT_209s0054g00530.1) are available in Genome Annotation Batch Download of Grape Genome Annotation Project (http://genomes.cribi.unipd.it/grape/). The sequences of ZmBBX1 (zma100147736), ZmBBX2(zma100281837), ZmBBX3 (zma100281289), ZmBBX4 (zma100281883), ZmBBX5 (zma100383826), ZmBBX6 (zma100281064), ZmBBX7 (zma100281114), ZmBBX8 (zma100193195), ZmBBX9 (zma100304421), ZmBBX10 (zma100282916), ZmBBX11 (zma100383648), ZmBBX12 (zma100283083), ZmBBX13 (zma100272654), ZmBBX14 (zma100283174), ZmBBX15 (zma100383388), ZmBBX16 (zma100273363), ZmBBX17 (zma100283103), ZmBBX18 (zma100284784), ZmBBX19 (zma100273793), ZmBBX20 (zma100274169), ZmBBX21 (zma100193074), ZmBBX22 (zma100284380), ZmBBX23 (zma100285359) are available in Genome Annotation Batch Download of maize Genome Annotation Project (http://www.maizesequence.org/index.html). The sequences of PtBBX1 (POPTR_266027), PtBBX2 (POPTR_831202), PtBBX3 (POPTR_837482), PtBBX4 (POPTR_737847), PtBBX5 (POPTR_562026), PtBBX6 (POPTR_737468), PtBBX7 (POPTR_836808), PtBBX8 (POPTR_658893), PtBBX9 (POPTR_419707), PtBBX10 (POPTR_752788), PtBBX11 (POPTR_569981), PtBBX12 (POPTR_852783), PtBBX13 (POPTR_846848), PtBBX14 (POPTR_243653), PtBBX15 (POPTR_774835), PtBBX16 (POPTR_851021), PtBBX17 (POPTR_247140), PtBBX18 (POPTR_564570), PtBBX19 (POPTR_868551), PtBBX20 (POPTR_782168), PtBBX21 (POPTR_820080), PtBBX22 (POPTR_741179), PtBBX23 (POPTR_721866), PtBBX24 (POPTR_648693), PtBBX25 (POPTR_550941), PtBBX26 (POPTR_801530), PtBBX27 (POPTR_804174), PtBBX28 (POPTR_855496), PtBBX29 (POPTR_1094062), PtBBX30 (POPTR_1081997), PtBBX31 (POPTR_827145), PtBBX32 (POPTR_562313), PtBBX33 (POPTR_594447), PtBBX34 (POPTR_571834), PtBBX35 (POPTR_758524), PtBBX36 (POPTR_568988), PtBBX37 (POPTR_582065), PtBBX38 (POPTR_563458), PtBBX39 (POPTR_567790) are available in Genome Annotation Batch Download of *Populus tomentosa* Genome Annotation Project (http://www.genome.ad.jp/kegg/). The sequences of OsBBX1 (Os01g0202500), OsBBX2 (Os02g0176000), OsBBX3 (Os02g0178100), OsBBX4 (Os02g0606200), OsBBX5 (Os02g0610500), OsBBX6 (Os02g0646200), OsBBX7 (Os02g0724000), OsBBX8 (Os02g0731700), OsBBX9 (Os03g0351100), OsBBX10 (Os03g0711100), OsBBX11 (Os04g0493000), OsBBX12 (Os04g0497700), OsBBX13 (Os04g0540200), OsBBX14 (Os05g0204600), OsBBX15 (LOC_Os06g01340), OsBBX16 (Os06g0152200), OsBBX17 (Os06g0264200), OsBBX18 (Os06g0275000), OsBBX19 (Os06g0298200), OsBBX20 (Os06g0654900), OsBBX21 (Os06g0661200), OsBBX22 (Os06g0713000), OsBBX23 (Os07g0667300), OsBBX24 (Os08g0178800), OsBBX25 (Os08g0249000), OsBBX26 (Os08g0536300), OsBBX27 (Os09g0240200), OsBBX28 (Os09g0509700), OsBBX29 (Os09g0527900), OsBBX30 (Os12g0209200) are available in Genome Annotation Batch Download of Rice Genome Annotation Project (RGAP: http://rice.plantbiol-ogy.msu.edu/). The sequences of MdBBX1 (MDP0000202669), MdBBX2 (MDP0000294359), MdBBX3 (MDP0000172036), MdBBX4 (MDP0000289278), MdBBX5 (MDP0000939920), MdBBX6 (MDP0000761905), MdBBX7 (MDP0000598183), MdBBX8 (MDP0000280947), MdBBX9 (MDP0000140460), MdBBX10 (MDP0000733075), MdBBX11 (MDP0000271388), MdBBX12 (MDP0000283949), MdBBX13 (MDP0000264228), MdBBX14 (MDP0000264845), MdBBX15 (MDP0000551876), MdBBX16 (MDP0000297093), MdBBX17 (MDP0000194889), MdBBX18 (MDP0000198531), MdBBX19 (MDP0000232355), MdBBX20 (MDP0000177126), MdBBX21 (MDP0000587860), MdBBX22 (MDP0000298804), MdBBX23 (MDP0000222881), MdBBX24 (MDP0000800387), MdBBX25 (MDP0000901915), MdBBX26 (MDP0000208320), MdBBX27 (MDP0000915501), MdBBX28 (MDP0000383112), MdBBX29 (MDP0000131980), MdBBX30 (MDP0000314259), MdBBX31 (MDP0000247810), MdBBX32 (MDP0000458656), MdBBX33 (MDP0000697407), MdBBX34 (MDP0000302297), MdBBX35 (MDP0000565292), MdBBX36 (MDP0000151848), MdBBX37 (MDP0000157816), MdBBX38 (MDP0000273201), MdBBX39 (MDP0000298575), MdBBX40 (MDP0000321380), MdBBX41 (MDP0000185616), MdBBX42 (MDP0000713113), MdBBX43 (MDP0000140484), MdBBX44 (MDP0000298635), MdBBX45 (MDP0000321180), MdBBX46 (MDP0000198072), MdBBX47 (MDP0000782323), MdBBX48 (MDP0000759984), MdBBX49 (MDP0000398010), MdBBX50 (MDP0000548690), MdBBX51 (MDP0000664576), MdBBX52 (MDP0000128581), MdBBX53 (MDP0000244238), MdBBX54 (MDP0000232445), MdBBX55 (MDP0000128008), MdBBX56 (MDP0000321735), MdBBX57 (MDP0000686172), MdBBX58 (MDP0000272743), MdBBX59 (MDP0000697109), MdBBX60 (MDP0000488955), MdBBX61 (MDP0000127949), MdBBX62 (MDP0000313949), MdBBX63 (MDP0000122414), MdBBX64 (MDP0000189746) are available in Genome Annotation Batch Download of Apple Genome Annotation Project (http://www.rosaceae.org/). The sequences of PbBBX1 (Pbr016562.1), PbBBX2 (Pbr023570.1), PbBBX3 (Pbr019957.1), PbBBX4 (Pbr036464.1), PbBBX5 (Pbr040252.1), PbBBX6 (Pbr022786.1), PbBBX7 (Pbr026954.1), PbBBX8 (Pbr038936.1), PbBBX9 (Pbr020281.1), PbBBX10 (Pbr013295.1), PbBBX11 (Pbr028831.1), PbBBX12 (Pbr022361.1), PbBBX13 (Pbr038976.1), PbBBX14 (Pbr042773.1), PbBBX15 (Pbr019591.1), PbBBX16 (Pbr015820.1), PbBBX17 (Pbr005884.1), PbBBX18 (Pbr020473.1), PbBBX19 (Pbr032616.1), PbBBX20 (Pbr034751.1), PbBBX21 (Pbr033352.1), PbBBX22 (Pbr011255.1), PbBBX23 (Pbr000255.1), PbBBX24 (Pbr022252.1), PbBBX25 (Pbr022252.1) are available in Genome Annotation Batch Download of Pear Genome Annotation Project (http://www.ebi.ac.uk/Tools/pfa/iprscan/). In addition, the sequences of SIBBX1 (Solyc02g089520.1), SIBBX2 (Solyc02g089500.2), SIBBX3 (Solyc02g089540.2), SIBBX4 (Solyc08g006530.2), SIBBX5 (Solyc12g096500.1), SIBBX6 (Solyc07g006630.2), SIBBX7 (Solyc12g006240.1), SIBBX8 (Solyc05g020020.2), SIBBX9 (Solyc07g045180.2), SIBBX10 (Solyc05g046040.1), SIBBX11 (Solyc09g074560.2), SIBBX12 (Solyc05g024010.2), SIBBX13 (Solyc04g007210.2), SIBBX14 (Solyc03g119540.2), SIBBX15 (Solyc05g009310.2), SIBBX16 (Solyc12g005750.1), SIBBX17 (Solyc07g052620.1), SIBBX18 (Solyc02g084420.2), SIBBX19 (Solyc01g110370.2), SIBBX20 (Solyc12g089240.1), SIBBX21 (Solyc04g081020.2), SIBBX22 (Solyc07g062160.2), SIBBX23 (Solyc12g005420.1), SIBBX24 (Solyc06g073180.2), SIBBX25 (Solyc01g110180.2), SIBBX26 (Solyc10g006750.2), SIBBX27 (Solyc04g007470.2), SIBBX28 (Solyc12g005660.1), SIBBX29 (Solyc02g079430.2) are available in locus search of Sol Genomics Network (SGN:https://www.sgn.cornell.edu) database, and CaBBX1 (Capana02g003201), CaBBX2 (Capana02g003200), CaBBX3 (Capana02g003199), CaBBX4 (Capana01g004030), CaBBX5 (Capana12g000414), CaBBX6 (Capana07g000030), CaBBX7 (Capana00g004028), CaBBX8 (Capana07g001114), CaBBX9 (Capana00g004489), CaBBX10 (Capana03g003558), CaBBX11 (Capana00g001486), CaBBX12 (Capana11g002294), CaBBX13 (Capana03g000377), CaBBX14 (Capana02g002620), CaBBX15 (Capana08g002625), CaBBX16 (Capana12g000659), CaBBX17 (Capana04g000266), CaBBX18 (Capana07g002062), CaBBX19 (Capana09g000394), CaBBX20 (Capana06g000735), CaBBX21 (Capana08g002611), CaBBX22 (Capana05g001195), CaBBX23 (Capana07g001588), CaBBX24 (Capana00g004911) can be downloaded from the Pepper Genome Platform (PGP: http://peppergenome.snu.ac.kr/download.php).
