# Correction: Genome-wide and expression analysis of B-box gene family in pepper

**DOI:** 10.1186/s12864-023-09835-y

**Published:** 2023-12-18

**Authors:** Jing Ma, Jia-xi Dai, Xiao-wei Liu, Duo Lin

**Affiliations:** Engineering Laboratory of Genetic Improvement of Horticultural Crops of Shandong Province, Key Laboratory of Horticultural Plant Genetic Improvement and Breeding of Qingdao, College of Horticulture, Qingdao Agricultural University, 700 Changcheng Road, Qingdao, 266109 China

**Correction: BMC Genomics 22, 883 (2021)**

**https://doi.org/10.1186/s12864-021-08186-w**

Following publication of the original article [1], it was reported that there was an error in Fig. 8C, which was a duplicate of Fig. 7D.

**Fig. 8:**
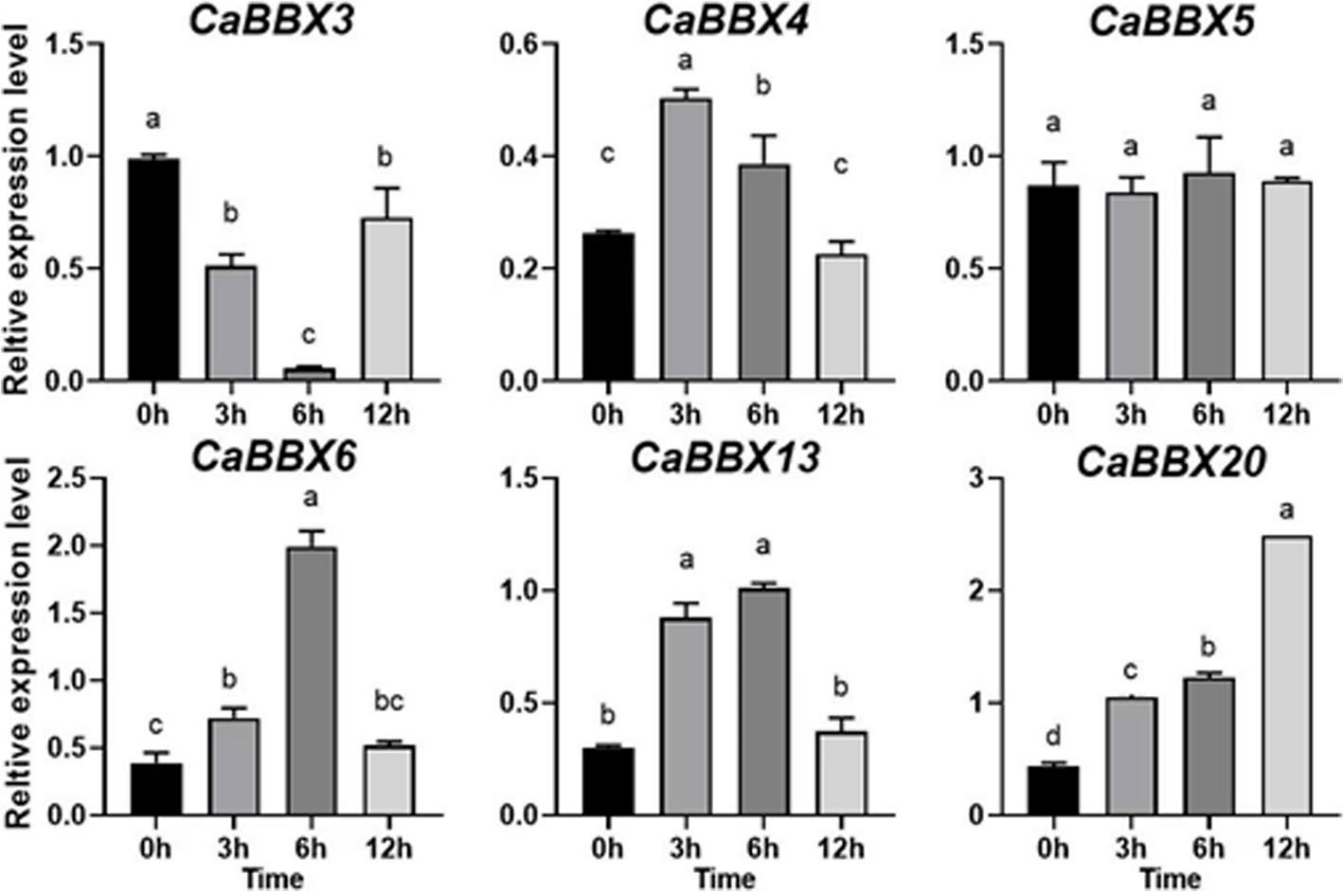
**C** qRT-PCR transcript analysis of 6 selected *CaBBX* genes under SA treatment. Three independent biological experiments were performed (*P* < 0.05)

Furthermore, the description of Fig. 8C in the section “Expression analysis of *CaBBX* genes in response to exogenous hormones” was incorrect.

- The incorrect description was: “*CaBBX13* was also up-regulated under early treatment stage, but down-regulated under later treatment stage (Fig. 8C).”
- The correct description is: “Except for *CaBBX3* and *CaBBX5*, all the other four selected *CaBBX* genes were induced to expressed at a higher level by exogenous SA at different treatment stages (Fig. 8C).”

In the section “Expression analysis of *BBX* genes under abiotic stress in pepper” the text describing Fig. 7D was also inaccurate:

- The incorrect description was: “Additionally, *CaBBX13* showed an up-regulation at 3 and 6 h, but rapidly decreased with a more lower expression level than control at 12 h treatment (Fig. 7D).”
- The correct description is: ““Additionally, *CaBBX13* showed an up-regulation at 6 h, but rapidly decreased with a more lower expression level than control at 12 h treatment (Fig. 7D).”

The correct Fig. 8C is given in this Correction article and the original article has been updated.
