# Supplementary material for: Genome-wide and expression analysis of B-box gene family in pepper
Source: BMC Genomics. 2021 Dec 6;22:883. doi: 10.1186/s12864-021-08186-w (PMC8650552; doi:10.1186/s12864-021-08186-w)
Supplement: Supplementary file 1 — Additional file 1: Table S1. Primer sequences used for real-time qRT-PCR amplification. [file 12864_2021_8186_MOESM1_ESM.docx]

| **Name** | **Gene ID** | **Primer sequences (5'-3')** | **Primer sequences (5'-3')** | **TM±SD** |
| --- | --- | --- | --- | --- |
| *CaBBX1* | Capana02g003201 | TGCTGTTGGTGGTAGATAA | GGGGAAGTAGTGGATGAC | 52.6±0 |
| *CaBBX2* | Capana02g003200 | ACTGGCACAACGCTATCT | TGGAGGGGACTGTCAATT | 52.85±2.90 |
| *CaBBX3* | Capana02g003199 | AACCCTTTATGGTCCTCCAGC | ATCATCCTCCGTGCCTTCC | 57.45±0.21 |
| *CaBBX4* | Capana01g004030 | TGGTTGCTACAAGCACCAAAT | CGTCGCCAAACAÀATACTCC | 54.55±1.20 |
| *CaBBX5* | Capana12g000414 | GTGCCGATTCTGCGTTTCT | ACACTCGCCGGAGCTTGTT | 56.25±1.48 |
| *CaBBX6* | Capana07g000030 | CCCATCAATCAGTGTGTCAT | CCCGGCAAATTTGGTAAATC | 53.4±0 |
| *CaBBX7* | Capana00g004028 | TTGTGAGTTGTCCTTTCC | TAGATGACCCTTCAGTCG | 51.45±1.63 |
| *CaBBX8* | Capana07g001114 | CCACATTGGGTAGGTCGT | TGAGCAGGGGATGGGTTC | 56.05±1.63 |
| *CaBBX9* | Capana00g004489 | AACTACCCAAAATCTCGTAACCAC | TATAACAAAGATCCGGCACCC | 55.9±0.42 |
| *CaBBX10* | Capana03g003558 | GAGATTGTTCTTCATTTGC | TCAGGTCCTAAAGGTTCA | 49.5±1.13 |
| *CaBBX11* | Capana00g001486 | GAGGCAGTAATGGAGTAAA | GATATTGAGGCCAAGAAA | 49.45±1.91 |
| *CaBBX12* | Capana11g002294 | CCTGCAACGCTGTCTCCTTAG | TGCCCATATTAGATCCCTTCG | 57.55±2.76 |
| *CaBBX13* | Capana03g000377 | TGGCAGTAGTAAAGCTGATG | CCATGGCGAAGATTGATTTG | 53.4±0 |
| *CaBBX14* | Capana02g002620 | AGGGCACAAAAGCATACA | GAATTAGCCAGGAAGACG | 51.45±1.63 |
| *CaBBX15* | Capana08g002625 | TGATTGTTGCCACTACGC | ACCAACCAAACAGGGAGA | 52.6±0 |
| *CaBBX16* | Capana12g000659 | GATGGATTGATTTGTGGG | TTTGAACTTTGGTGGACA | 49.2±1.56 |
| *CaBBX17* | Capana04g000266 | CTTCTGAGGAGGTGGAGC | GAGATGTTACCTGGATGGC | 56.2±1.41 |
| *CaBBX18* | Capana07g002062 | AGGAGCAAAATCCCCAAC | CGAAATGCCCCAGTATCA | 52.6±0 |
| *CaBBX19* | Capana09g000394 | CGCTGTAGTTGAAGGCGATGA | AGCTGTTGGTGGCGAAGAA | 56.4±1.70 |
| *CaBBX20* | Capana06g000735 | ATGCCTCCCAACCACTCAAA | CCTCAGCAGAATACCCAACAAG | 56.45±1.77 |
| *CaBBX21* | Capana08g002611 | TTGCAAAAGGTGTCGCAATC | AGAGGGTTCTGTTTCAACTGGTAG | 55.65±3.18 |
| *CaBBX22* | Capana05g001195 | TACGGACCGATGAAGCTGTT | ATGCCTGATGTTGATTTGACCT | 54.7±0.99 |
| *CaBBX23* | Capana07g001588 | CGGTCCTTGTATCCTCGT | AATGCGTGAAACAAATGC | 51.5±4.81 |
| *CaBBX24* | Capana00g004911 | CAACCCGAACCACAAAGACG | GCATAGCGAGGAAGGTAAAGG | 57.5±014 |
| *Actin* |  | GGTGACGAGGCTCAATCCAA | CTCTGGAGCCACACGAAGTT | 57.45±0 |
