# Supplementary figures and images for: Genome-wide and expression analysis of B-box gene family in pepper
Source: BMC Genomics. 2021 Dec 6;22:883. doi: 10.1186/s12864-021-08186-w (PMC8650552; doi:10.1186/s12864-021-08186-w)

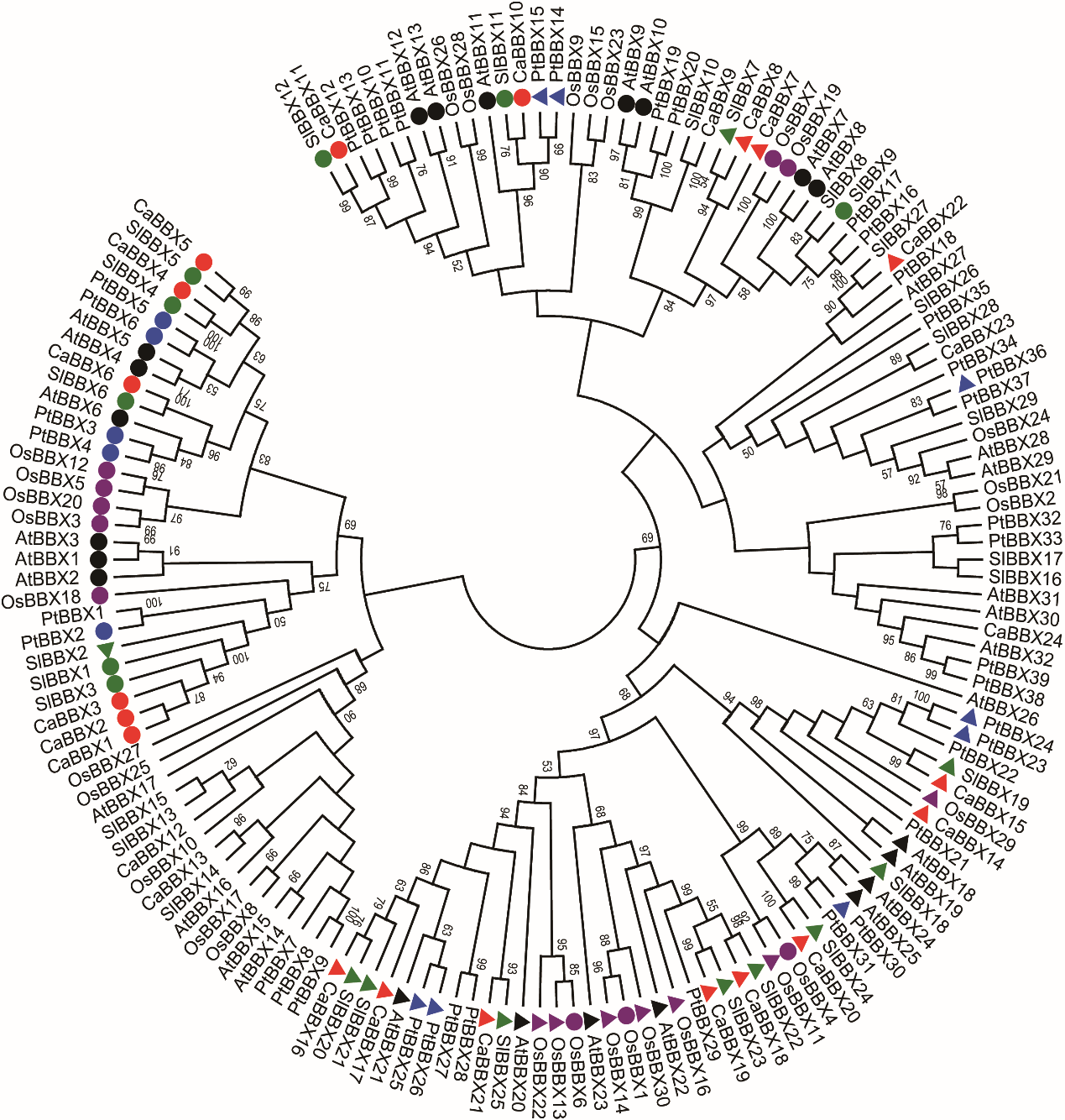

Supplement: Supplementary file 2 — Additional file 2: Figure S1. Phylogenetic tree of BBX members from Capsicum annuum, Solanum lycopersicum, Arabidopsis thaliana, Oryza sativa and Populus trichocarpa (Ca represent Capsicum annuum; Sl represent Solanum lycopersicum; At represent Arabidopsis thaliana; Os represent Oryza sativa; Pt represent Populus trichocarpa). The members marked in circle contain two B-BOX and one CCT domains, and in triangle contain two B-BOX domains (red represent Capsicum annuum; green represent Solanum lycopersicum; black represent Arabidopsis thaliana; purple represent Oryza sativa; blue represent Populus trichocarpa). [file 12864_2021_8186_MOESM2_ESM.docx]

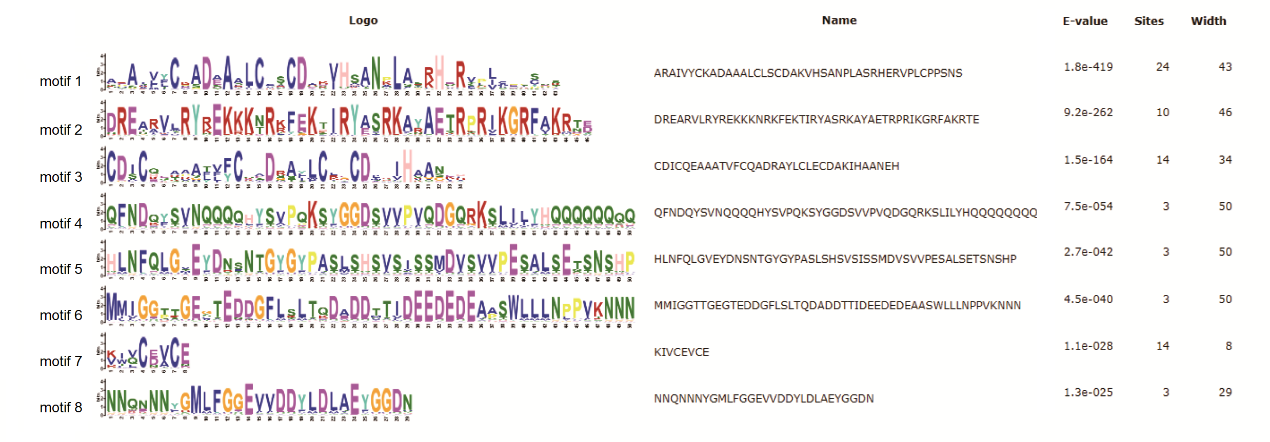

Supplement: Supplementary file 3 — Additional file 3: Figure S2. Amino acid arrangement order of motif of BBX family. [file 12864_2021_8186_MOESM3_ESM.docx]

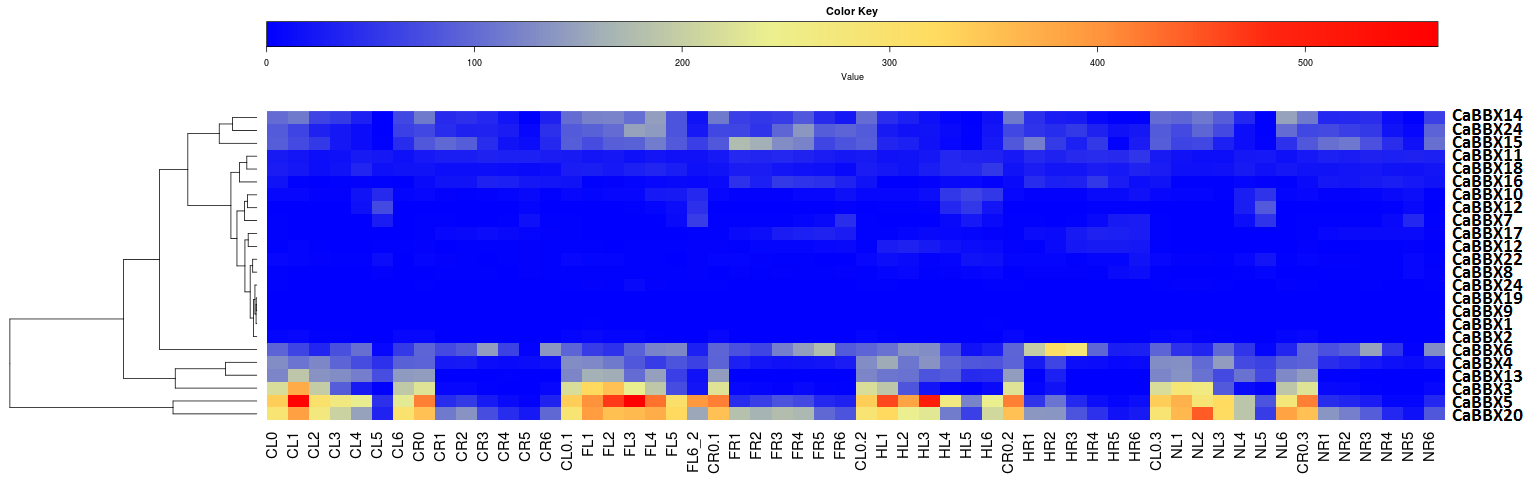

Supplement: Supplementary file 4 — Additional file 4: Figure S3. Expression profiles of CaBBX genes under different abiotic stresses. The first character C, F, H and N of the abbreviation on the bottom represent control, freezing, heat and NaCl treatment, respectively; the second character L and R represent leaf and root, respectively; the number 0, 0.1 and 0.2 represent 0h treatment; and the number 1, 2, 3, 4, 5 and 6 represent 1h, 1.5h, 3h, 6h, 12h and 24h treatment. [file 12864_2021_8186_MOESM4_ESM.docx]

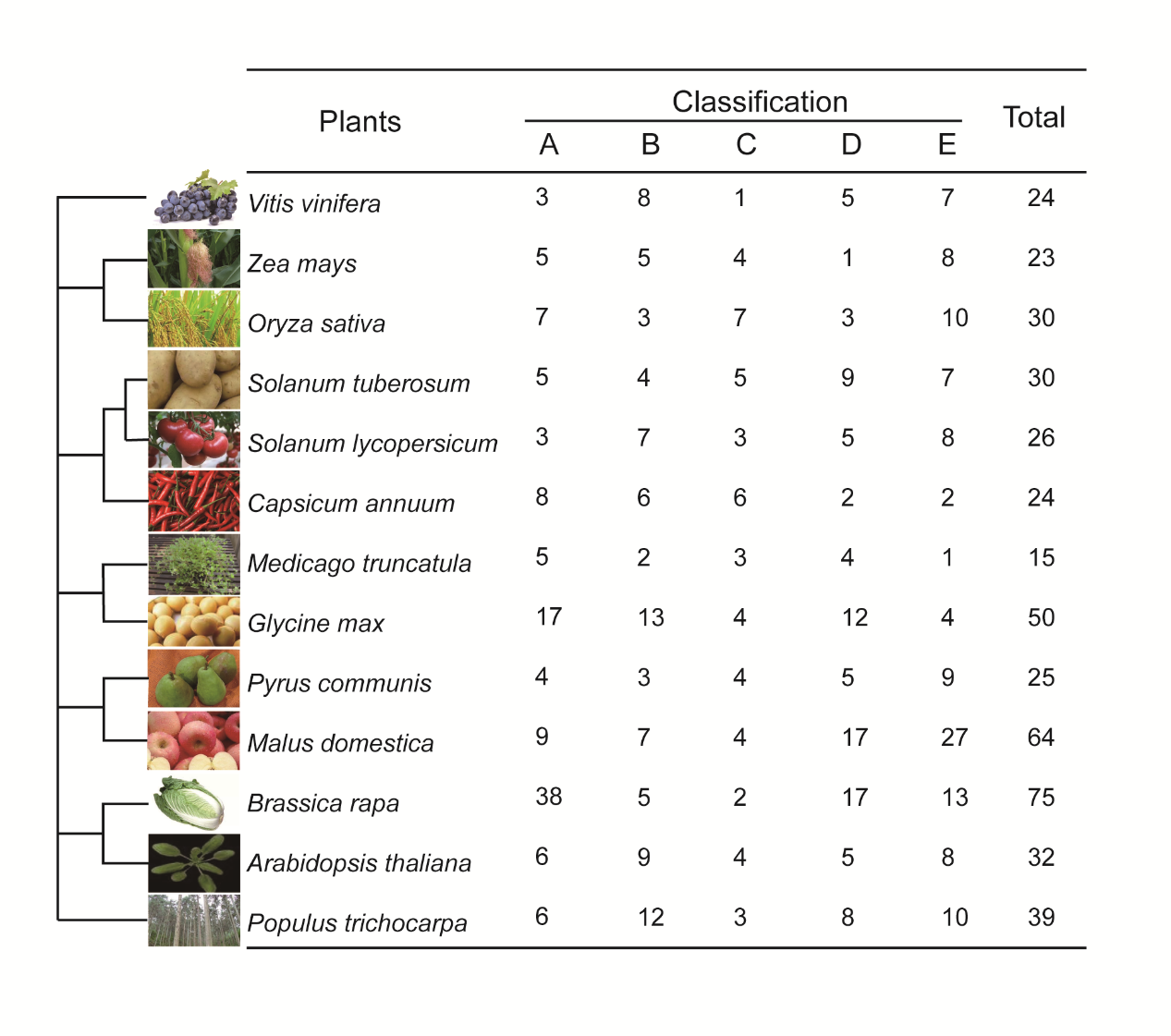

Supplement: Supplementary file 5 — Additional file 5: Figure S4. The classification of BBX genes investigated in 13 higher plants (A-E representative five different subclasses in the BBXs Groups I ~ V, respectively). [file 12864_2021_8186_MOESM5_ESM.docx]
